# Tomato *short internodes and pedicels* encode an LRR receptor-like serine/threonine-protein kinase ERECTA regulating stem elongation through modulating gibberellin metabolism

**DOI:** 10.3389/fpls.2023.1283489

**Published:** 2023-11-17

**Authors:** Xueya Zhao, Kunpeng Zhang, Huidong Zhang, Mengxi Bi, Yi He, Yiqing Cui, Changhua Tan, Jian Ma, Mingfang Qi

**Affiliations:** ^1^ College of Horticulture, Shenyang Agricultural University, Shenyang, China; ^2^ National & Local Joint Engineering Research Center of Northern Horticultural Facilities Design & Application Technology (Liaoning), Shenyang, China; ^3^ Key Laboratory of Protected Horticulture (Shenyang Agricultural University), Ministry of Education, Shenyang, China; ^4^ Key Laboratory of Horticultural Equipment, Ministry of Agriculture and Rural Affairs, Shenyang, China; ^5^ Collaborative Innovation Center of Protected Vegetable Provincial Co-construction Surrounds Bohai Gulf Region, Shenyang, China

**Keywords:** tomato, short internodes, short pedicels, ERECTA, gibberellin

## Abstract

Plant height is an important agronomic trait. Dwarf varieties present several advantages, such as lodging resistance, increased yield, and suitability for mechanized harvesting, which are crucial for crop improvement. However, limited research is available on dwarf tomato varieties suitable for production. In this study, we report a novel short internode mutant named “*short internode and pedicel (sip*)” in tomato, which exhibits marked internode and pedicel shortening due to suppressed cell elongation. This mutant plant has a compact plant structure and compact inflorescence, and has been demonstrated to produce more fruits, resulting in a higher harvest index. Genetic analysis revealed that this phenotype is controlled by a single recessive gene, *SlSIP*. BSA analysis and KASP genotyping indicated that *ERECTA* (*ER*) is the possible candidate gene for *SlSIP*, which encodes a leucine-rich receptor-like kinase. Additionally, we obtained an *ER* functional loss mutant using the CRISPR/Cas9 gene-editing technology. The 401^st^ base A of *ER* is substituted with T in *sip*, resulting in a change in the 134^th^ amino acid from asparagine (N) to isoleucine (I). Molecular dynamics(MD) simulations showed that this mutation site is located in the extracellular LRR domain and alters nearby ionic bonds, leading to a change in the spatial structure of this site. Transcriptome analysis indicated that the genes that were differentially expressed between *sip* and wild-type (WT) plants were enriched in the gibberellin metabolic pathway. We found that GA_3_ and GA_4_ decreased in the *sip* mutant, and exogenous GA_3_ restored the *sip* to the height of the WT plant. These findings reveal that *SlSIP* in tomatoes regulates stem elongation by regulating gibberellin metabolism. These results provide new insights into the mechanisms of tomato dwarfing and germplasm resources for breeding dwarfing tomatoes.

## 1 Introduction

Ideal plant architecture is a significant breeding objective for numerous crops (Reinhardt and Kuhlemeier, 2002). Dwarfism is a desirable characteristic in crop breeding because plant biomass is strongly correlated with its height, confers enhanced resistance to lodging damage from wind and rain, and is associated with stable and increased yields by improving the harvest index (Law et al., 1978; Miralles et al., 1998; Li et al., 2018). In particular, in the 1960s and 1970s, the Green Revolution, which introduced semi-dwarf wheat and rice varieties, substantially increased grain yield throughout Asia (Peng et al., 1999; Khush, 2001). Presently, a considerable number of dwarfing genes have been identified, characterized, and successfully cloned in diverse crop species. For instance, more than 60 dwarfing genes have been identified and cloned in rice, including *sd-1*, *D-h*, and *OsKS2* (Liu et al., 2017). In wheat, 26 dwarfing genes have been identified, primarily belonging to the *Rht* family (Wang et al., 2014). In tomatoes, genes associated with gibberellin and brassinosteroid synthesis and signaling pathways, such as *PROCERA*, *JMJ524*, and *DWARF*, have been identified to regulate plant height (Bassel et al., 2008; Li et al., 2015; Livne et al., 2015; Li et al., 2016). Cavasin et al. (2021) evaluated a tomato line carrying the dwarf gene and reported a yield gain of 8.04%. Many dwarf and semi-dwarf cultivars, such as Micro-Tom and Micro-Gold, have been developed as ornamental cultivars or used in genetic transformation studies because of their greater spatial efficiency (Scott et al., 1995; Meissner et al., 1997; Gerszberg et al., 2014). Dwarf breeding is a beneficial option for adjusting different crops to a particular cropping system without compromising productivity or other traits. However, dwarf tomato varieties suitable for crop production have yet to be explored and developed.

Several dwarfing mutants are accompanied by shortening of other tissues and organs. Pedicels are specialized internodes that support and orient flowers and fruits at an upward angle on the inflorescence stem (Douglas et al., 2002). The length of the pedicel is related to traits, such as mechanized harvesting. Additionally, the length and orientation of pedicels directly determine the architecture of the inflorescence or spikes, which can significantly affect crop yield (Prenner et al., 2009). A single *ERECTA* (*ER*) mutation in *Arabidopsis thaliana* resulted in short stature and compact inflorescences (Torii et al., 1996). The *ER* family (*ERf*) is considered a crucial signal in plant development, among the key players during morphogenesis, and generates different responses in different tissues and organs (Shpak, 2013). *ERECTA* family of receptors is an ancient family of leucine-rich repeat RLKs that contain a signal peptide, leucine-rich repeat in the extracellular domain, transmembrane domain, and cytoplasmic serine/threonine protein kinase domain (Lease et al., 2001). Previous research on *ER* mutants in *Arabidopsis thaliana* has revealed that *ER* is one of the most important genes for promoting localized cell proliferation. *ER* controls multiple aspects of plant growth and development, such as stomatal development and differentiation (Shpak et al., 2005), plant morphogenesis, inflorescence morphology, leaf initiation (DeGennaro et al., 2022), multiple organ differentiation, and drought resistance (Shen et al., 2015; Li et al., 2021).

The function and mechanism of *ERfs* are best comprehended in *Arabidopsis thaliana* concerning stomatal development. ERf receptors activity are regulated by the secretion of peptides, including those of the EPF/EPFL family, which can function as agonists or antagonists during stomatal development (Shpak, 2013). Ligands that bind to the LRR domain can activate the ER protein and subsequently trigger the mitogen-activated protein kinase (MAPK) signal transduction cascade involving YDA, MKK4/MKK5, and MPK3/MPK6 (Meng et al., 2012). The ER kinase domain is relatively conserved across species, whereas the extracellular LRR domain is markedly different (Kosentka et al., 2017). This suggests that the ER-MAPK module exhibits a relatively stable behavior, and that mutations in the LRR domain are crucial for investigating ER function. The mechanism by which ER regulates stem cell development is similar to that of stomata. *ERfs* are expressed in the epidermis and xylem, and EPF/EPFL act as upstream regulators of ER activity (Uchida et al., 2012), whereas the MAPK cascade functions as a downstream regulator of ER signaling (Meng et al., 2012). *ER* expression under a series of promoters can rescue elongation defects of stems, pedicels, epidermis, and leaves when expressed in the phloem (Uchida et al., 2012). EPFL4 (also known as CHALLAH-LIKE2 [CLL2]) and EPFL6 (CHALLAH [CHAL]) are upstream of *ERfs* and redundantly regulate stem elongation and inflorescence growth. The *epfl4 epfl6* double mutant does not have any apparent epidermal defects but shows dwarfing and compact inflorescences (Abrash et al., 2011). PACLOBUTRAZOL RESISTANCE (PRE) is downstream of the MAPK cascade, which promotes pedicel elongation (Cai et al., 2017). HOMOLOG OF BEE2 INTERACTING WITH IBH 1(HBI1) is downstream of PRE1. HBI1 can promote the expression of the BR biosynthesis genes *CYP85A2* and *AUXIN RESPONSE FACTOR 3* (*ARF3*) by directly binding to their promoters, which influences the expression of genes involved in auxin biosynthesis and signaling. Although the process of signal transmission from the extracellular to the intracellular space is complex, its ultimate outcome is determined by hormone signals. In melons, *ER* regulates stem elongation through auxin signaling by directly affecting polar auxin transport (Yang et al., 2020). Xu et al. (2023) found that an *ER* mutant in cucumber led to the formation of short internodes by reducing the expression of auxin genes and decreasing endogenous auxin content. However, the regulatory processes in tomatoes remain unclear.

In this study, we aimed to identify and functionally characterize a novel dwarf and short pedicels mutant named “*short internode and pedicel* (*sip*)” in tomato. Through BSA analysis, we found that the mutation in *sip* was due to the base substitution A134T in *ERECTA*, which encodes an LRR receptor-like serine/threonine protein kinase. This mutation induces conformational changes in the LRR domain, resulting in up-regulation of gibberellin degradation genes and changes in endogenous gibberellin levels. These findings will help to elucidate the mechanism of dwarfing in tomatoes and enable the development of potential dwarf-breeding genes.

## 2 Materials and methods

### 2.1 Plant materials and population development

Sequenced tomato ‘Heinz 1706’ as wild-type (WT) and ‘Alisa Craig’(AC) as parentage of another population were obtained from *Tomato Genetics Resource Center* (https://tgrc.ucdavis.edu/). *sip* is the mutant line of ‘Heinz 1706’ that has been self-crossed for six generations through ethyl methanesulfonate (EMS) mutagenization. For genetic analysis, *sip* was crossed with WT to obtain the F1 and F2 generations. The F1 was backcrossed with the parents to obtain BC1 generations. Similarly, *sip* was crossed with AC to obtain F1, F2, and BC1 progeny. All plants were grown in greenhouses at 15–28 °C at the Shenyang Agriculture University(Shenyang, China).

### 2.2 Microscopic observation of internode and pedicel

The central elongating sections of the stems of the WT and *sip* plants were sampled at the seedling stage with six true leaves. The stalks sampled at the flowering stage contained out-of-zone parts from the WT and *sip* plants for comparison of their microscopic structures. Tissues were fixed overnight in formalin-acetic alcohol (FAA). The samples were then dehydrated in a gradient alcoholic solution and soaked in different proportions of ethanol-xylene solution. These tissues were embedded in paraffin, and transverse and longitudinal sections were stained with toluidine blue and examined under a Zeiss microscope (MicroBeam IV, Germany).

### 2.3 Pool construction and bulked-segregant analysis

We used two F2 populations to identify *SlSIP*: cross *sip* × WT for MutMap and cross *sip* × AC for QTL-seq. For MutMap analysis, we used 28 F2 individuals (WT-*sip* hybrid population) with shortened internodes and pedicels to construct the DNA pools “F2dw-pool”. For QTL-seq, 45 individuals with short internodes and flower stalks were selected from the isolated population of AC and *sip* hybrid in the F2 generation to construct “SF-pool”. In comparison, 50 individuals with normal height and long pedicels were selected to construct “HL-pool”. Genomic DNA from the parents (WT, AC, and *sip*) and F2 was extracted from fresh leaves using a DNA Secure Plant Kit (Tiangen, Beijing, China).

Qualified total genomic DNA was used to construct paired-end libraries with an insert size of 300–400 bp using a Paired-End DNA Sample Prep kit (Illumina Inc., San Diego, CA, USA). These libraries were sequenced using a NovaSeq6000 (Illumina Inc., San Diego, CA, USA) at GeneDenovo (Guangzhou, China). To identify SNPs and InDels, the BWA software (Li and Durbin, 2009) was used to align the clean reads from each sample to the tomato reference genome SL4.0 (https://solgenomics.net/organism/Solanum_lycopersicum/genome). The analytical method was the same as that described by (Wang et al., 2017). The QTL-seq method was referenced and improved on the method proposed by Takagi et al. (2013). Metadata for the bioproject are available at NCBI under the accession number PRJNA1006521 and PRJNA1007367.

### 2.4 SNP genotyping by KASP and PARMS

According to the SNPs between the WT and *sip* identified via DNA sequencing (MutMap). Kompetitive allele-specific PCR (KASP) markers in the 20 and 40 Mb regions of chromosome 8 (chr.8) that are specific for different subgenomes were designed and named M20 and M40, respectively. Based on mutations in the candidate genes *Solyc08g061560* and *Solyc08g062600*, we designed markers K1 and K2 for genotyping. For genotyping analysis, DNA was extracted from 3 WT, 3 *sip*, and 165 F2 individuals showing the *sip* phenotype.

The penta-primer amplification refractory mutation system (PARMS) is a KASP-like SNP genotyping technique that combines ARMS, also known as allele-specific PCR (Heim and Meyer, 1990), with universal energy transfer–labeled primers (Nuovo et al., 1999). Based on the QTL-seq results, we designed markers every 5 Mb within a range of 10–50 Mb on chromosome 8. These markers were named P1–P10. SNP calling and plotting were performed using the online software snpdecoder (http://www.snpway.com/snpdecoder/) with manual modifications. All primer sequences are listed in **Supplementary Table S1**.

### 2.5 CRISPR/Cas9Pubi-H-ER vector construction and tomato genetic transformation

The two SG sequences used were SG61560-1: 5’-GCTGATTACTGTGCCTGGAG-3’ and SG61560-2: 5’-GGGGAGTTGTCTCCTGCTAT-3’. Oligo dimer was prepared using a 20-µL reaction mixture containing 16 µL of annealing buffer, 1 µL of SG61560-UP1, 1 µL of SG61560-LW1, 1 µL of SG61560-UP2, and 1 µL of SG61560-LW2. The reaction mixture was centrifuged at 12000 rpm for 1 min and was diluted to 10 µM by adding deionized water. The reaction conditions were denaturation at 95 °C for 3 min and annealing. The oligo dimer was inserted into the target vector in a reaction mixture containing 1 µL of saCas9/g RNA Vector, 1 µL of oligo dimer, 1 µL of Solution1, 1 µL of Solution2, and 6 µL of H_2_O. It was incubated at 16 °C for 2 h. The sequencing primer used was PUV4-R: 5′-TCCCAGTCACGACGTTGTAA-3′. The transgenic tomato lines were selected based on their hygromycin resistance. Tomato genetic transformation method can be referred to Sun et al. (2020).

### 2.6 Molecular dynamics simulation of ER and SlSIP

The protein sequences of ER and SlSIP were obtained from NCBI (*National Center for Biotechnology Information* https://www.ncbi.nlm.nih.gov/). RosetaTTAFold (Baek et al., 2021) was used for modeling, and confidence scores were 0.81 and 0.78, respectively. A good-quality model was used for the subsequent analyses. Gromacs2019.6 (Van Der Spoel et al., 2005) was selected as the kinetic simulation software and amber14sb was selected as the protein force field. A TIP3P water model was applied to the system, a water box was established (with its edge at least 1.2 nm from the protein edge), and a sodium ion balance system was added. Particle-mesh Ewald (PME) handles electrostatic interactions using the steepest descent method for energy minimization over 50,000 steps. The cutoff distances of the Coulomb force and van der Waals radius were 1.4 nm. A canonical ensemble (NVT) and constant-pressure and constant-temperature (NPT) were used to balance the system. Subsequently, 100 ns MD simulations were performed at normal temperature and pressure. The V-rescale temperature coupling method and Berendsen (Berendsen et al., 1984) method were used to control the simulated temperature at 300 K and pressure at 1 bar. The results were analyzed using various built-in Gromacs package functions, such as root mean square deviation (RMSD), radius of gyration (RoG), root mean square fluctuation (RMSF), and solvent-accessible surface area (SASA). PyMOL 2.5.1 (Mooers, 2019) was used for the graphical display and LigPlot 2.1 (Laskowski and Swindells, 2011) was used for the visual display.

### 2.7 RNA extraction and transcriptome sequencing

Approximately 500 mg of the third internode of the WT and *sip* was collected at four true leaf stage for transcriptome analysis. Total RNA from six samples (three biological replicates per sample) was extracted using TRIzol Reagent (Invitrogen, Carlsbad, CA, USA). The RNA-seq analysis was performed as described previously (Sun et al., 2020). Metadata for the bioproject are available at NCBI under the accession number PRJNA1003223.

### 2.8 Real-time quantitative PCR

Stems from 50 WT and *sip* plants were collected from 3–4 true leaves for mRNA extraction using an RNA extraction kit (Kangwei, China) and cDNA synthesis using reverse transcriptase (TaKaRa, China). The tomato gene *SlActin* was used as a control (all primer sequences are listed in **Supplementary Table S2**). A 20-μL reaction mixture was prepared including 2 µL of cDNA (1:10 dilution), 10 µL of 2× TransStart Top Green qPCR SuperMix (TransGen, China), 0.5 µL of each primer (10 µmol/µL), and ddH_2_O. The reaction conditions were: 95°C for 7 min; 40 cycles at 95°C for 10 s, 58°C for 30 s, and 72°C for 20 s; and then 71 cycles at 95°C for 10 s, 60. 5°C for 10 s, and 95°C for 10 s. The relative gene expression levels were calculated using the 2^−ΔΔCt^ method (Livak and Schmittgen, 2001).

### 2.9 Plant hormone treatment and endogenous GA determination

Seedlings at the 3–4 leaf stage of *sip* and WT were treated with foliar sprays of GA_1_ (100 µM), GA_3_ (100 µM), GA_4_ (100 µM), GA_7_ (100 µM), IAA (50 µM), and H_2_O (as a control). Spraying was performed every three days for one week, and plant height was recorded. Sampling was performed, during the four-leaf stage, approximately two weeks after the seeds were planted. Approximately 1 g of young and tender stems (specifically the second internode) was collected from both *sip* mutants and WT plants, with three biological replicates for each. Phytohormones were quantified following Li et al. (2019) with slight modifications, using a liquid chromatography-mass spectrometry system (Agilent 1290, AB company Qtrap6500; California, CA, USA).

### 2.10 Subcellular localization

The coding sequence (CDS) of *ER* was cloned into the binary vector pCAMBIA-1300-35S-GFP. Primer sequences: LRR-1300-BAMI-F:5’-GCTCGGTACCCGGGGATCCATGGCATCATTTTTACTCCAAAGAT-3’ (Tm: 60.8 °C); and LRR-1300-SALI-R: 5’-GCCCTTGCTCACCATGTCGACGCCACTATTCTGGGATATGACCT-3’ (Tm: 59.3 °C). The recombinant constructs were transformed into *Agrobacterium tumefaciens* strain GV3101. Subsequently, transformed material was cultured in YEP liquid medium (containing 50 mg·mL^-1^ kanamycin and 100 mg·mL^-1^ rifampicin) at 28°C for 16 h. The bacteria were then centrifuged and slowly resuspended in a suspension medium (10 mM MES, 10 mM MgCl_2_, 1 M acetosyringone). After 48h of incubation in the dark, the fluorescence signals were observed using a Leica laser confocal microscope (TCS SP82400301, Germany).

### 2.11 *In situ* hybridization

The unique sequence of the *ER* transcript was amplified from WT cDNA using ExTaq polymerase (TaKaRa) and ligated into pSPT18 and pspt19 vectors. The constructs were linearized and subjected to RNA labeling reaction using T7 or SP6 RNA polymerase. Slice were prepared and *in situ* hybridization analysis was performed following the methods described by Cheng et al. (2022).

### 2.12 Prediction of promoter binding elements

The promoter sequences (2000 bp before the coding region) of genes related to gibberellin synthesis and degradation were obtained from NCBI. The promoter sequences were then predicted using the online promoter prediction software PlantCare (http://bioinformatics.psb.ugent.be/webtools/plantcare/html/) and PlantRegMap (http://plantregmap.gao-lab.org/binding_site_prediction_result.php).

## 3 Results

### 3.1 Phenotypic identification and genetic analysis of short internodes and pedicels mutants *sip*

**Figure 1:**
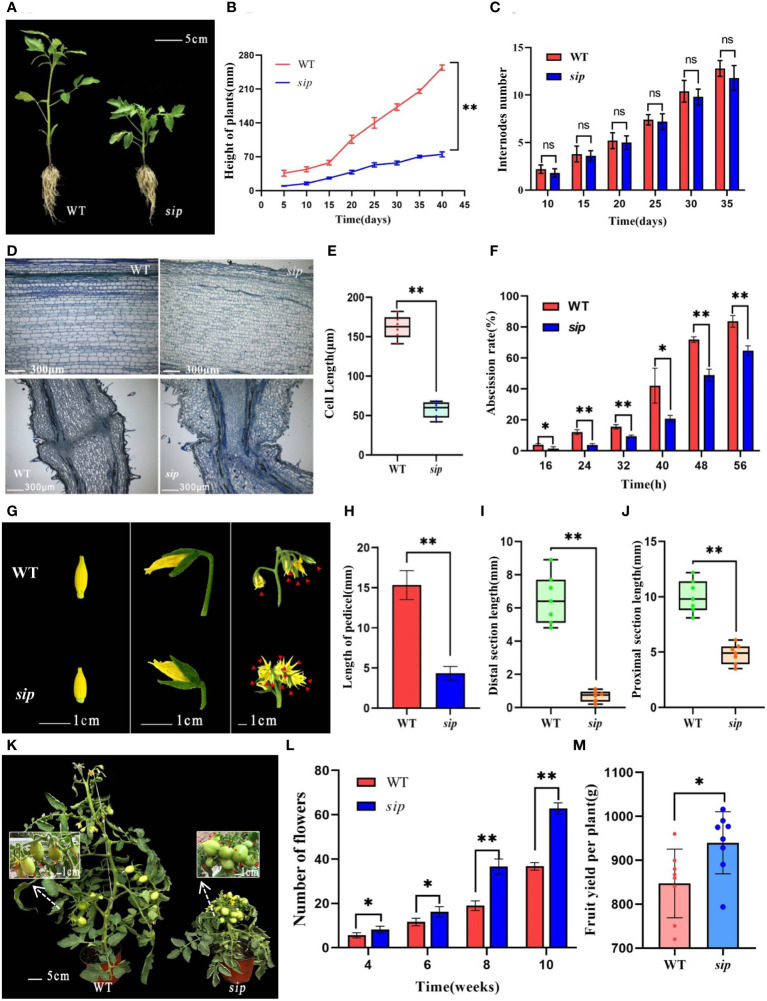
Phenotypic identification of *sip* mutants. **(A)** Plant height of WT and *sip* at five true leaves stage (n=3). **(B, C)** Plant height and stem internode number of two tomato lines at different stages (the beginning of sowing is regarded as 0 days, n=3). **(D)** Microscopic observation of longitudinal sections of stems and peduncles in WT and *sip*(the third internode of WT and *sip* at five true leaves stage and pedicels at the stage of full petal expansion). **(E)** Length of stem cells. **(F)** Abscission rate of WT and *sip* (three replicates, with 50 flower stalks per replicate). **(G)** Morphology of inflorescences and peduncles in WT and *sip* (flowers within the inflorescence marked with red arrows). **(H–J)** Length of the pedicels and lengths of the proximal section and distal section in WT and *sip* (n=7). **(K)** Plant morphology and fruit-setting capacity at the fruiting stage (fruits marked with red arrows). **(L)** Statistical analysis of the number of flowers in WT and *sip* plants. **(M)** Yield per plant. Student’s t-test was used to determine a significant difference. *p < 0.05 and **p < 0.01; ns, no significant difference. All data are presented as mean ± SD.

In this study, Heinz ‘1706’ was used as WT. Additionally, a stably inheritable dwarf mutant line “*sip*,” exhibiting significantly shortened internodes and petioles, was obtained through EMS mutagenesis. From the seedling stage, *sip* showed significant dwarfism compared to WT (**Figure 1B**), which persisted throughout the growth period (**Figures 1A, B, K**). At the fifth true leaf stage, the plant height of the *sip* was only 58% that of the WT (**Figures 1A, B**). However, the number of internodes in *sip* and WT remained constant throughout the different periods (**Figure 1C**), and have the same number of leaves during the seedling stage (**Supplementary Figure 1A**), suggesting that dwarfing was due to internode shortening rather than a growth lag. Observation of paraffin sections of stems indicates that the elongation of stem cells are significantly inhibited in *sip* (**Figures 1D, E**), but the cross-section showed no significant difference in cell area (**Supplementary Figures 1B, C, H**). In addition to internode shortening, the most prominent phenotype of *sip* was a compact inflorescence structure with severely shortened flower stalks (**Figure 1G**). The shortening stalk was caused by both the shortening of the proximal axial end length and the abnormal development of the position of the abscission zone, which made the distal axial end almost disappear (**Figures 1G–J**). Considering the abnormal morphology of the abscission zone, we compared the abscission rates of WT and *sip* and found that *sip* had a lower abscission rate than that of WT. Paraffin sections of petioles revealed that the small cell layer of the abscission zone of *sip* was discontinuous between the medulla and cortex compared with that of the WT (**Figures 1D, F**), which might explain why *sip* was less likely to have abscission than the WT. *sip* exhibits a compact cluster of inflorescences, interestingly, the single inflorescence of *sip* can form more flowers (**Figures 1G, M**).This characteristic enables *sip* to produce more fruits, resulting in a significantly higher yield index (**Figures 1K, M**; **Supplementary Figure 1L**). We have noticed that compared to WT, the fruits of *sip* have a more flattened round shape, and the ratio of fruit diameter to length has decreased, as well as the weight of individual fruits (**Supplementary Figures 1D-G, K**). However, this does not affect the yield per plant of *sip*, as it has more fruits.

To genetically characterize the short internodes with the loss-abaxial pedicel phenotype of the *sip* mutant, we generated an F2 population of 716 individuals by crossing the WT with *sip*. All F1 plants showed normal plant height and pedicels. Specifically, 533 individuals out of 716 from the F2 population displayed a normal phenotype similar to the WT, whereas the remaining 183 individuals were similar to *sip* with a 3:1 segregation ratio, as revealed by the chi-square test (**Table 1**, χ^2^ = 0.09). This indicates that a single recessive gene is responsible for *sip* phenotype. This hypothesis is further supported by the 1:1 Mendelian ratio observed in the BC1F1 population (**Table 1**; χ^2^ = 0.17).

**Table 1:** Genetic analysis of the *sip* phenotype in different populations.

Generations	Total	High	dwarf with short pedicel	Segregation ration	*x* ^2^
P_1_(WT)	50	50	0		
P_2_(*sip*)	50	0	50		
F_1_(P_1_×P_2_)	54	54	0		
F_1_(P_2_×P_1_)	58	58	0		
BC_1_(F_1_×WT)	49	49	0		
BC_1_F_1_×*sip*	53	25	28	0.89:1	0.17
F_2_	716	533	183	2.91:1	0.09

x^2^_0.05,1_ = 3.84.

### 3.2 Mapping of the *SlSIP* by BSA-seq

**Figure 2:**
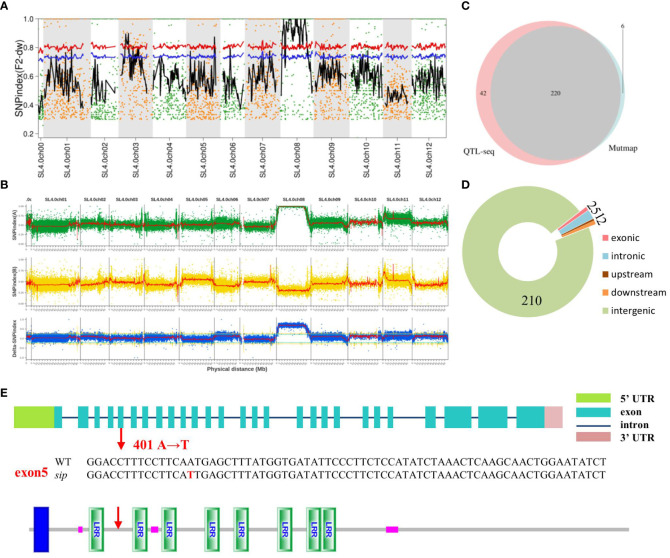
Genome-wide distribution of SNP-index. **(A)** Differences in allele frequencies among 28 lines with the *sip* phenotype in the F2 population and WT (the red and blue line represents the 99% and 95% confidence interval, respectively). **(B)** Differences in allele frequencies between 45 lines with the *sip* phenotype in the F2 population and AC. The X-axis represents the 12 chromosomes of tomato. The Y-axis represents the difference in allele frequencies (SNP values) between the two pools. **(C)** The Venn diagram of SNP-index of the two databases. **(D)** The distribution of SNPs in the intersection of the two databases. **(E)** Analysis of CDS sequence and protein sequence structure of mutation site in *sip*. (Red arrows represent mutation sites; protein structure prediction using online prediction tool: http://smart.embl-heidelberg.de/).

Candidate genes were identified using MutMap analysis. In total, 181746682 and 175293598 clean reads (150 + 150 bp) were acquired from the F2*sip*-pool (average read depth > 34.27×depth coverage or 99.84% coverage) and the WT (33.05×depth coverage or 99.83% coverage), respectively. To reduce background noise, we used the sliding window method to fit the SNP index and related calculation results. The allele frequency differences of 4,233 filtered SNPs from the two pools were calculated and mapped across the 12 tomato chromosomes to create a Manhattan plot (**Figure 2A**). Only the 3^rd^ and 8^th^ chromosomes had SNPs with confidence intervals exceeding 99%. Surprisingly, a large number of SNP-index peak values of 1 in chr.8 were observed. After excluding the F2-*sip* heterozygous sites and sites congruent with the reference genome, we obtained 226 SNPs on chr.08. Two SNPs were found in exons, six in introns (two upstream and two downstream), and the remaining 214 in intergenic regions. Candidate genes were defined based on 12 single nucleotide polymorphisms (SNPs) located outside the intergenic region (**Table 2**).

**Table 2:** List of candidate genes.

Candidate Genes	Pos	Ref	Alt	Function Type	Position
*Solyc08g061560*	47106679	T	A	nonsynonymous SNV	exon5:401
*Solyc08g062600*	49603202	G	A	nonsynonymous SNV	exon1:343
*Solyc08g013840*	3204201	G	T	–	intron3:84
*Solyc08g048250*	14004482	G	A	–	intron5:890
*Solyc08g022130*	31865227	G	A	–	intron3:342
*Solyc08g028690*	41250405	G	A	–	intron1:8357
*Solyc08g061500*	46933486	G	A	–	intron23:21
*Solyc08g065870*	52310931	G	A	–	intron3:1444
*Solyc08g015900*	6189225	C	T	–	upstream dist:976
*Solyc08g066120*	52648757	G	A	–	upstream dist:1735
*Solyc08g047990*	14912391	C	A	–	downstream dist:781
*Solyc08g044280*	21869137	G	A	–	downstream dist:381

Upstream: region 2 kb upstream of the transcription start site. Downstream: The 2 kb region downstream of the transcription termination site.

To obtain fewer candidate genes and shorter candidate regions, another F2 generation isolated population was constructed by crossing *sip* with the distantly derived variety (AC). The F2 generation had a 3:1 separation ratio, 304 tall plants with normal pedicels, and 121 short internodes with loss-abaxial plants (χ2 = 0.09). The shortened internodes at the seedling stage and the structure of the absent flower stalk made it easy to identify extreme phenotypes in the F2-isolated populations. QTL-seq was used and 282.43 Gb of clean data were obtained by Illumina NovaSeq, including 30.47 Gb, 29.25 Gb, 104.04 Gb, and 118.67 Gb from the AC, *sip*, F2SF-pool, and F2HL-pool mixed pools, respectively, all of which were of high quality (94.56% > Q30 > 92.84%) and had stable GC content (37.14% > GC > 36.45%). The average sequencing depths for the parent and F2 pools were 32.09× and 110.04×, respectively. A total of 293,701 SNPs were obtained from the parents and two mixed pools. The result positioned *SlSIP* to a large interval on chromosome 8 (**Figure 2B**). Although the candidate region remains extensive, the sequencing results from QTL-seq indicated that of the 17,713 SNPs on chr.8, 10,427 SNPs with differences were found to be in concordance with the reference genome. The 6,138 heterozygous loci in *sip* were also disregarded because it was previously determined that the mutated genes were recessive. Since there were no dwarfing or short pedicel plants in the parent AC under normal cultivation conditions, SNPs that exhibited the same genotype as *sip* in AC could also be excluded. After excluding heterozygous SNPs and those that were consistent with the reference genome in the F2SF-pool, and then removing SNPs in the F2HL-pool that had the same genotype as *sip*, 262 remaining SNPs were identified as potential candidates. The SNP distribution and annotation statistics are presented in **Supplementary Table S3**.

We developed PARMS markers every 5 Mb between 10 Mb and 50 Mb on chromosome 8 and validated them in F2 individuals with *sip* phenotype, yielding consistent results indicating significant linkage of large segments (**Supplementary Figure 2**). To narrow the range of candidate genes, we compared the data from the two sequencing results. Interestingly, we observed a substantial overlap (**Figure 2C**), which further confirmed the accuracy of both the sampling and sequencing processes. The overlapping portions of the two results included two SNPs located in the exonic region, five SNPs in the intronic region, one in the upstream region, two in the downstream region, and 210 in the intergenic region (**Figure 2D**).

### 3.3 Screening and identification of candidate genes as *ERECTA*

Candidate genes were present in the shared regions between the two sequencing results, and we temporarily ignored SNPs in the intergenic regions. Mutations in both exons were nonsynonymous. *Solyc08g061560* encodes an LRR receptor-like serine/threonine-protein kinase (ERECTA), and *Solyc08g062600* is an *Exostosin family-like protein probable arabinosyltransferase ARAD1*. We analyzed SNPs located in introns and none at intron boundaries (**Table 2**). Mutations in the upstream region may result in changes in the transcription recognition sites that alter gene expression at the transcriptional level. There may be some lncRNA recognition sites in downstream regions that regulate gene transcription. Additional quantitative fluorescence verification showed that only *Solyc08g044280* expressed in the stems of seedlings among these three genes and the mutation did not affect their transcription at the seedling stage (**Supplementary Figure 3**).

These results suggest that the two nonsynonymous mutations in this exon may be candidate genes for the *sip* phenotype. Thus, we designed the KASP probe marking and classification to determine whether the two genes had a mutation phenotype separation. According to the WT and *sip* hybrid F2 populations, 92 individuals had short internodes and flower stalks for genotyping. Only SNP with *ERECTA* mutations were co-isolated from the mutant phenotype (**Supplementary Table S4**). These results indicated that *ERECTA* is a candidate gene responsible for the *sip* phenotype. Sequencing results showed a specific alteration was induced in exon 5 of ERECTA, resulting in the replacement of 401^st^ base of the CDS from A to T (**Figure 2E**). This alteration resulted in the substitution of amino acid 134 from N to I. This amino acid change results in the conversion of the polar hydrophilic side chain into a nonpolar hydrophobic side chain. This suggests that the spatial conformation of the receptor was modified to some extent.

### 3.4 Protein structure analysis of the LRR domains of ER and SlSIP by MD simulations

**Figure 3:**
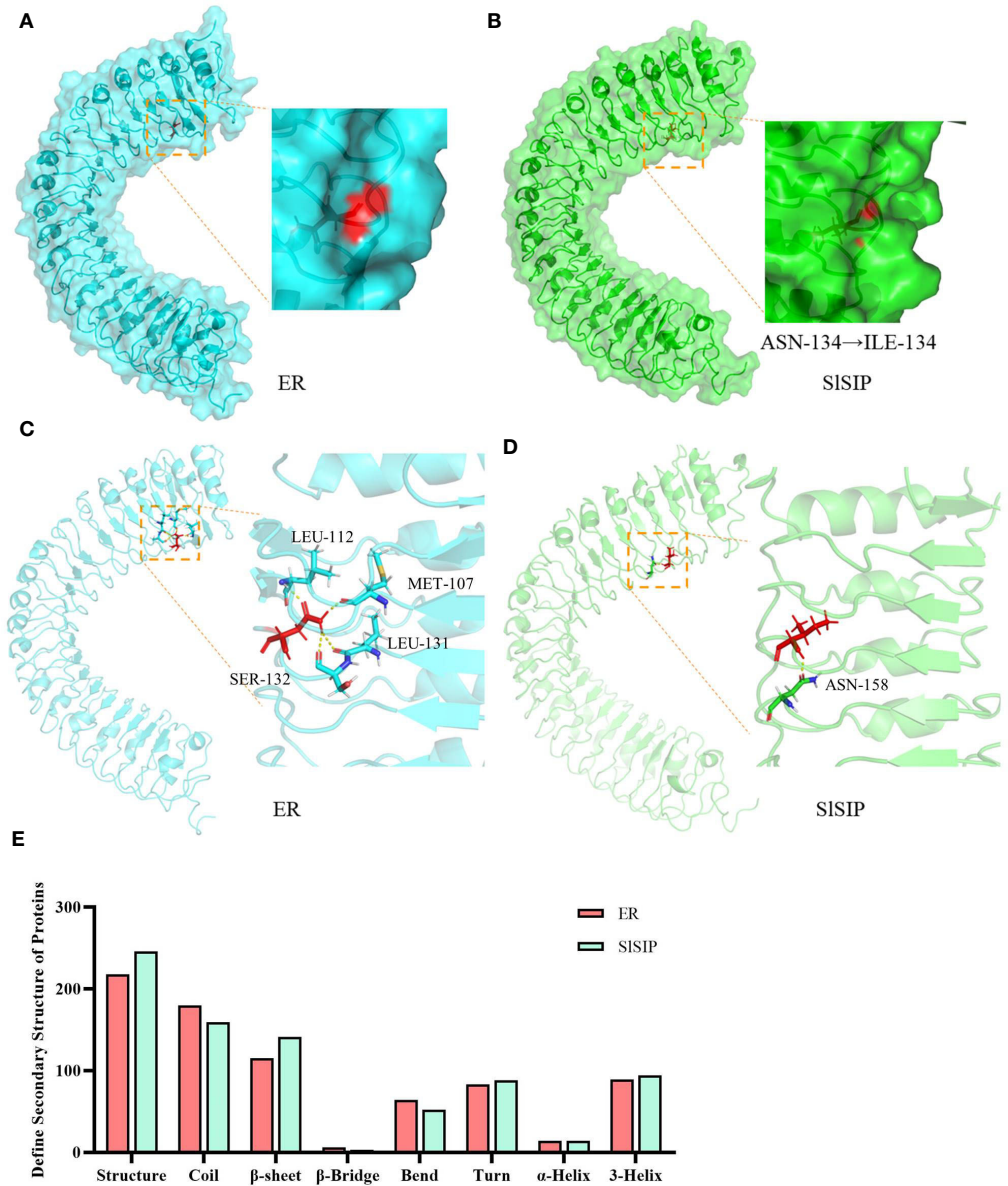
Protein structures of the LRR structural domains from ER and SlSIP by MD simulations. **(A, B)** The surface structure of the ER and SlSIP proteins, with 134 amino acid highlighted in red. **(C, D)** The rigid structure and internal binding of the ER and SlSIP proteins; the 134^th^ amino acid marked in red, and the amino acids that form hydrogen bonds with the 134^th^ residue are marked in different colors. **(E)** The differences in secondary structure between two LRR domains.

The overall structure remained rigid after mutation, with an RMSD of 1.05 nm (**Figures 3A–D**, **Supplementary Figure 4**). Further examination of the mutated amino acids in the stick format revealed that ASN^134^ and ILE^134^ were located in the coil region of the overall structure. Specifically, they were positioned at the interface between the interior and exterior of the structure. Notably, Asn^134^ was more exposed than Ile^134^, indicating a higher degree of surface accessibility (**Figures 3A, B**). In particular, ASN^134^ in the extracellular region of a protein can interact with other amino acids. For the WT protein, hydrogen bonds were observed between Asn^134^ and Met^107^, Leu^112^, Leu^131^, and Ser^132^, all of which were located in parallel coil structures (**Figure 3C**). However, only contact with Asn^158^ has been observed at the mutant site Ile^134^ (**Figure 3D**). Calculations and simulations revealed differences in the secondary structures under stable conditions (**Figure 3E**). When comparing horizontally, we found that the mutated structures, β-sheets, turns, and 3-Helix, increased, whereas coils and bends decreased. In summary, this mutation significantly disrupts the original internal contacts of the protein, leading to increased flexibility and decreased rigidity. This alteration results in the emergence of new secondary protein structures.

### 3.5 Function verification of *SlSIP*

**Figure 4:**
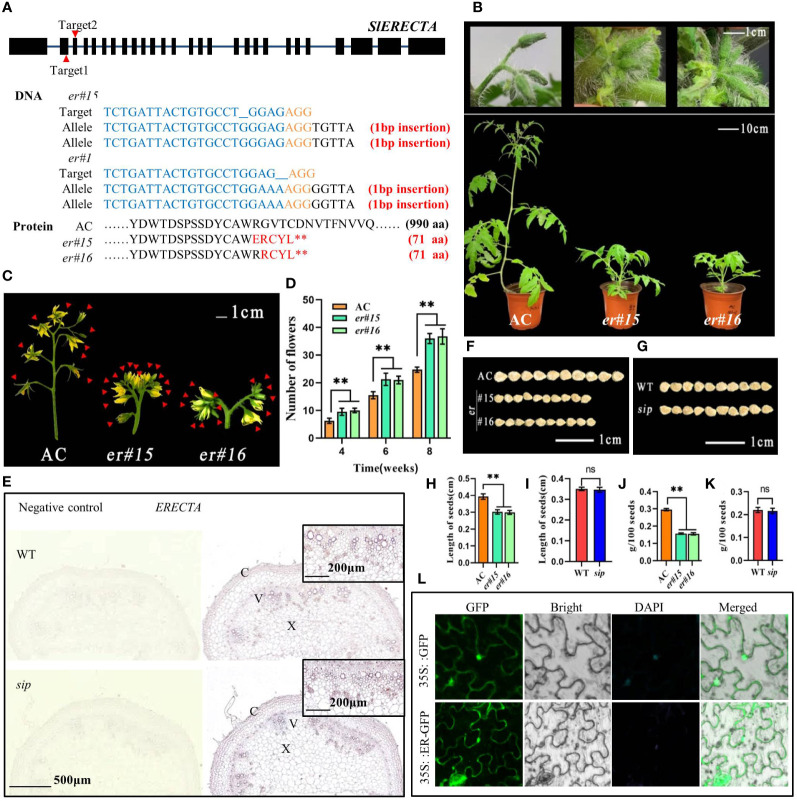
Functional verification of candidate gene *SlSIP*. **(A)** Targeting information of CRISPR/Cas9Pubi-H-ER vector and sequence alignment of *ER* mutant and background material. **(B)** Comparison of the phenotypes of *ER* loss-of-function mutants *er#15* and *er#16* with AC. **(C)** Comparison of inflorescences between AC and *er* mutant (flowers marked with red arrows). **(D)** Statistical analysis of the number of flowers in AC and *er* plants. **(E)** *In situ* hybridization of *ER* expression in the stems of WT and *sip*. **(F, G)** Comparison of seed size. **(H–K)** Length of seed (n=10) and hundred-seed weight (n=3). **(L)** Subcellular localization of ER. Student’s t-test was used to determine a significant difference. **P < 0.01; ns, no significant difference. All data are presented as mean ± SD.

To validate the function of *SlSIP*, we deleted the *ER* gene from the AC background using the CRISPR/Cas9 technology. The specific locations of the two target sites are shown in **Figure 4A**. After performing sequencing analyses on 30 plants in the T0 generation, we confirmed the presence of two homozygous *er* mutant plants, *er#15* and *er#16*, and obtained the T1 generation. Non-edited negative plants were used as controls. Notably, plant *er#15* and *er#16* underwent precise editing at target site 1, resulting in a 1-bp deletion that caused the formation of a premature stop codon (**Figure 4A**). Consistent with the phenotype of *sip*, *er#15* and *er#16* both show obvious internode shortening, compact inflorescence, and shortened pedicels (both proximal section length and distal section length are significantly reduced), but the number of internodes has not decreased (**Figure 4B**; **Supplementary Figures 5A–F**). Compared to AC, the number of flowers in each inflorescence in *er* mutants have also increased, and correspondingly, the mutants produce more fruits per inflorescence (**Figures 4C, D**; **Supplementary Figures S5G, S6**). Similar to *sip*, *er* mutants also undergo changes in fruit morphology, with no significant difference in fruit diameter compared to AC, but a decrease in fruit length leads to a decrease in the fruit index and fruit weight (**Supplementary Figures S5H–L**). This consistent phenotype further supported the hypothesis that *SlSIP* functions as *ER*.

Subcellular localization findings indicate that ER in tomato is positioned on the cell membrane (**Figure 4L**). *In situ* hybridization results showed that, similar to other species, *ER* was highly expressed in vascular bundles. However, there was no significant difference in the expression of *ER* in *sip* stems compared to that in the WT (**Figure 4E**). This indicated that the short internodes in *sip* stems were not caused by a decrease in *SlSIP* expression. Additionally, we have discovered an intriguing phenomenon. We compared the *sip* and *ER* knockout mutants and found that some *ER* functions were unaffected in *sip*. Specifically, we found that the seeds of the *er* mutant plants from both strains displayed a significant size reduction compared to that of AC (**Figure 4F**) and exhibited a significant decrease in seed length and 100-seed weight (**Figures 4H, J**). However, this phenomenon was not observed in the WT and *sip* (**Figures 4G, I, K**). Intriguingly, we also found that the disruption of ER function in tomatoes had a direct impact on seed size., suggesting that the mutated domain in *sip* plays a specific role in regulating stem elongation in tomato.

### 3.6 ER regulates internode elongation by modulating gibberellin levels in tomatoes

**Figure 5:**
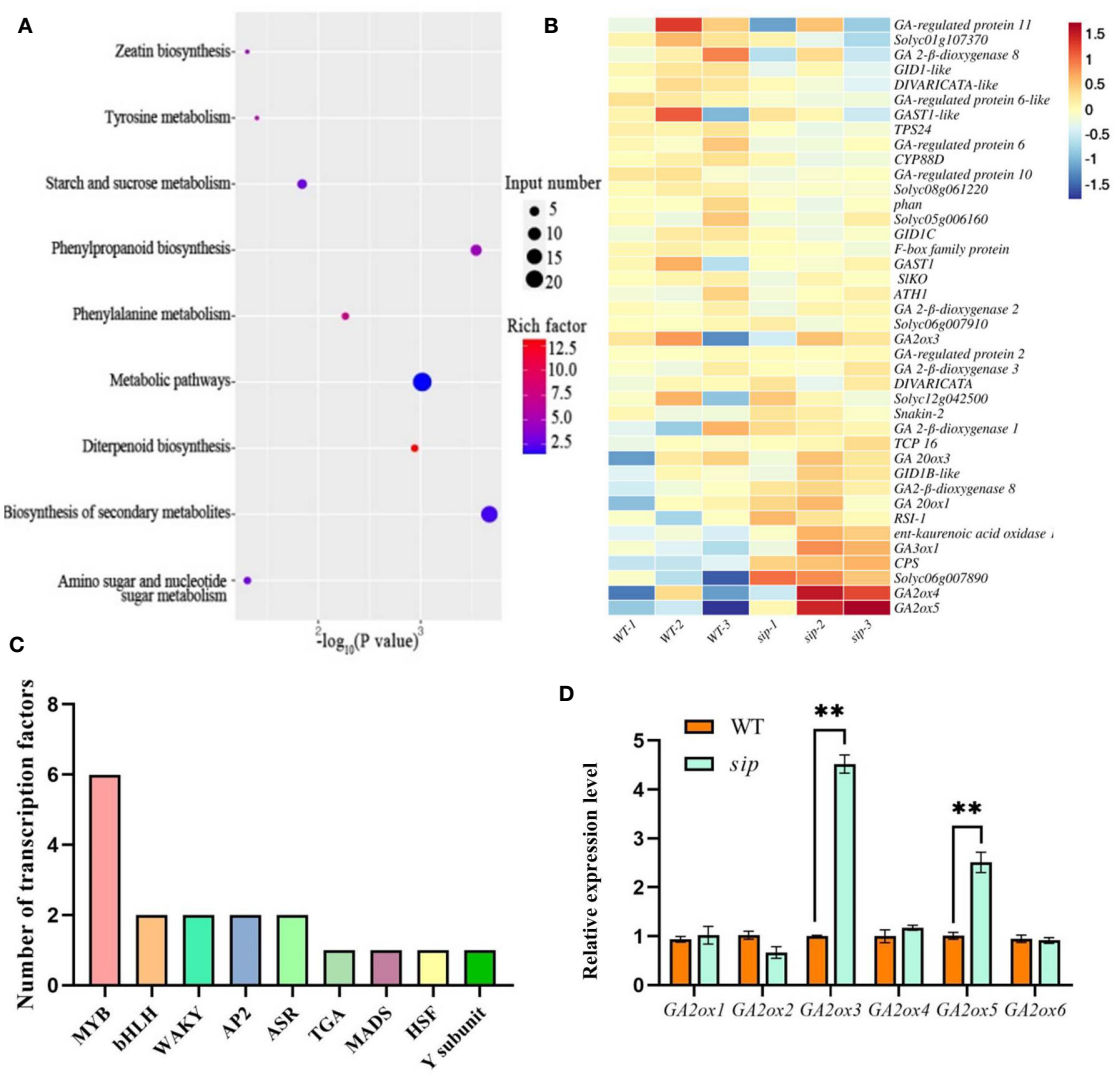
Differentially expressed genes between WT and *sip*. **(A)** KEGG pathway map of up-regulated genes. **(B)** Heat map of gibberellin-related genes in RNA-seq. **(C)** Number of differentially expressed transcription factors. **(D)** Real-time quantitative validation of differentially expressed genes (n=3). A Student’s t-test indicated a significant difference. **P < 0.01. All data are presented as mean ± SD.

To gain insight into the mechanism by which ER regulates stem elongation, total mRNA was extracted from the young and delicate stems of WT and *sip* plants for transcriptome profiling. High-throughput sequencing was performed on 6 RNA-seq libraries, with three replicates of WT obtaining 52,976,758, 44,569,918, and 45,661,190 clean reads, and *sip* obtaining 45,345,936, 47,858,102, and 51,281,884 clean reads, respectively. A total of 18,504 genes were expressed in the young stems. Using a threshold of log_2_FC absolute value > 1 and p value < 0.05, 86 genes were down-regulated and 184 genes were up-regulated in expression (**Supplementary Figure 7A**, **Supplementary Table S5**). Cluster analysis demonstrated a high level of consistency between the three biological replicates of both WT and *sip* (**Supplementary Figure 7B**). To analyze the functional significance of these differentially expressed genes (DEGs), Gene Ontology (GO) term enrichment analyses and KEGG pathway enrichment analysis were conducted to investigate the potential biological functions of the DEGs. GO enrichment analysis showed that they were mainly enriched in three categories: biological process, cellular component, and molecular function (**Supplementary Figure 8**). KEGG pathway enrichment analysis reveals that up-regulated differentially expressed genes are mainly enriched in diterpenoid biosynthesis pathways, phenylalanine metabolism and RNA polymerase, etc. KEGG pathway map of The down-regulated genes are mainly enriched in various metabolic pathways such as linoleic acid metabolism and diterpenoid biosynthesis pathways (**Figure 5A**; **Supplementary Figure 9**, **Supplementary Table S6**). Several transcription factor expression levels are changed in the *sip* variant juvenile stems, with 11 up-regulated and 7 down-regulated, including 6 members of the MYB family, 2 members of the bHLH family, 2 members of the WARK family, 2 members of the AP2 family, 2 members of the ASR family, 1 member of the TGA family, 1 member of the HSF family, 1 member of the MADS family, and Nuclear Transcription Factor Y subunit B-5-like (**Figure 5C**).

**Figure 6:**
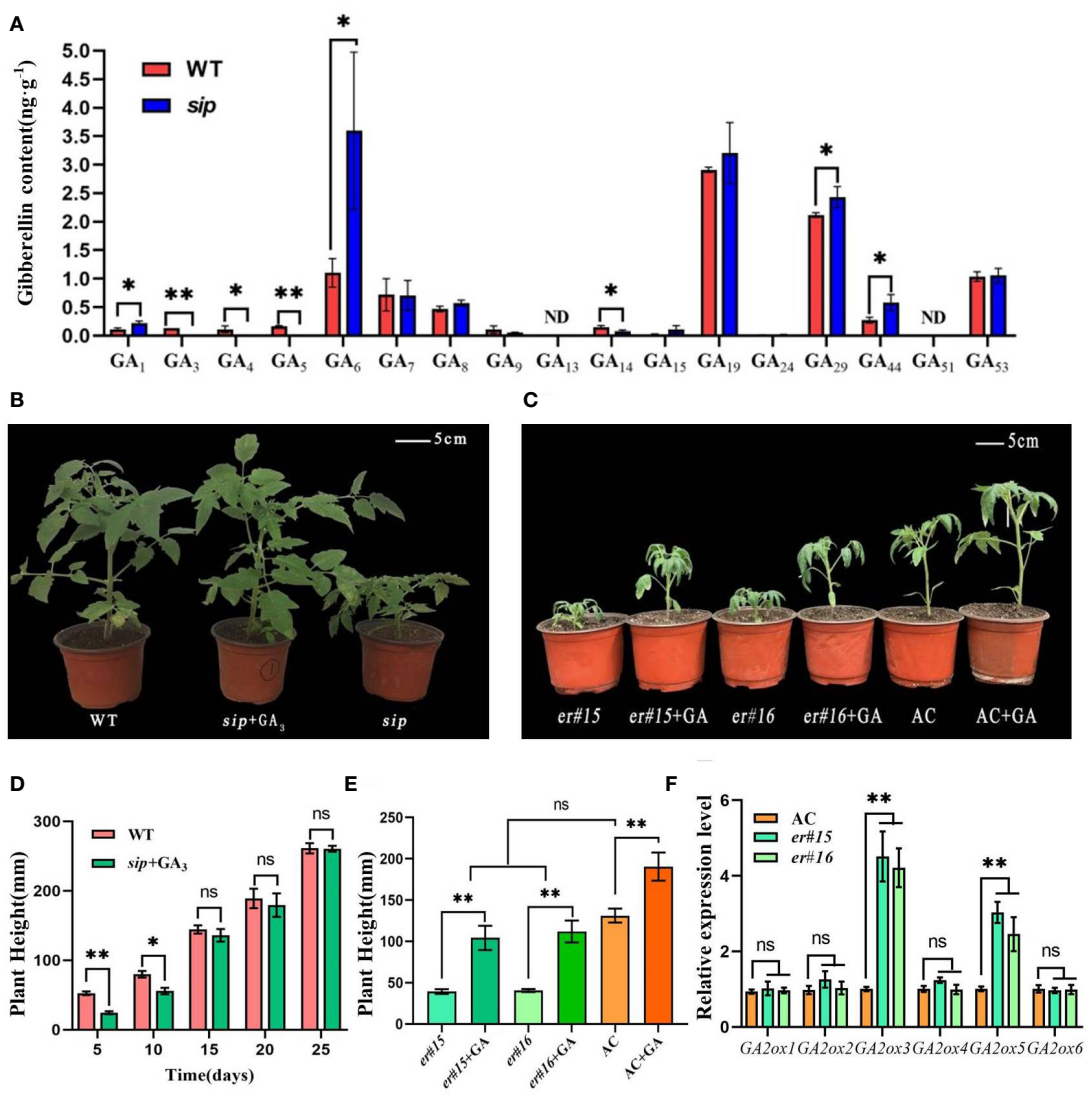
Gibberellin content and hormone recovery experiments. **(A)** Gibberellin content in stems of WT and *sip* (n=3). **(B, D)** Exogenous application of gibberellin restores the height of *sip* to that of WT. **(C, E)** Exogenous application of gibberellin restores the height of *er* to that of AC. **(F)** Expression of *GA2oxs* in the stems of *er* mutant. Student’s *t*-test was used to determine significance. *p < 0.05 and **p < 0.01; ns, no significant difference. All data are presented as mean ± SD.

Diterpenoids are precursors of gibberellin synthesis, and several DEGs have been implicated in the gibberellin signaling pathway, including gibberellin biosynthesis, degradation, and polar transport genes (**Figure 5B**). Quantitative real-time PCR analysis verified that the relative expression levels of gibberellin degradation genes were significantly higher in *sip* than in WT (**Figure 5D**). Is the short internode of sip caused by the up-regulated expression of gibberellin-degrading genes? We further measured the gibberellin content in the young stems of both WT and *sip*. Measurement of endogenous gibberellin levels revealed a significant reduction in the levels of the active gibberellins GA_3_ and GA_4_ in *sip* (**Figure 6A**). Exogenous application of GA_3_ restored the height of *sip* to that of WT plants, and *ER* knockout mutants *er#15* and *er#16* responded similarly to gibberellin (**Figures 6B–E**). Quantitative real-time PCR result reveals that the expression levels of *GA2oxs* are elevated in the stems of both *er#15* and *er#16* (**Figure 6F**). These lines of evidence suggest that *ER* may regulate internode elongation in tomatoes by modulating gibberellin metabolism.

## 4 Discussion

### 4.1 Identification of a novel dwarf mutant in tomato

Dwarfing varieties possess significant characteristics such as being space-saving, being resistant to lodging, and being suitable for mechanized harvesting. Most commercial tomato varieties have long stems and many lateral branches, necessitating a considerable amount of time and labor for pruning. The tomato mutant we identified, *sip*, exhibits a compact phenotype reminiscent of a bouquet, while effectively reducing flower pedicel shedding and producing more fruit. These unique traits not only make it suitable for mechanized harvesting but also hold potential for its utilization as an ornamental variety. We constructed two populations for mapping and analyzed the BSA sequencing results using bioinformatics. The findings from both populations exhibited a high degree of similarity. Both BSA results and the PRAMS markers suggested the presence of a significant linked region on chr.8, which makes the mapping of *sip* challenging. In this case, a comparison of the BSA results of the two populations proves to be highly valuable. We identified only two SNPs located within the exons that had the potential to contribute to the *sip* phenotype. To verify this, KASP genotyping was performed, and the results showed that only *SlSIP* co-segregated with the short internode and pedicel phenoty. Subsequent validation of *SlSIP*’s function was carried out with *ER* loss-of-function mutants created through CRISPR/Cas9 gene-editing technology, demonstrating that *SlSIP* was responsible for the *sip* phenotype.

*SlSIP* encodes a leucine-rich repeat receptor-like kinase ER, which is involved in regulating multiple aspects of plant growth and development. In *Arabidopsis*, the homologous gene *AtERECTA* exhibits similar phenotypic characteristics to *sip* and confers compact inflorescence, blunt fruits, and short petioles (Torii et al., 1996; Shpak et al., 2004; Meng et al., 2012; Uchida et al., 2012; Du et al., 2018). The reports on ER in tomato also support the aforementioned mapping results in terms of phenotype (Villagarcia et al., 2012; Kwon et al., 2020). Interestingly, when compared to *ER* signaling mutants in tomatoes that were created by expressing the dominant-negative, signal disrupter, truncated *ERECTA* (*AtΔKinase*) from *Arabidopsis* (Villagarcia et al., 2012), we did not observe any difference in stomatal density and flowering time in *sip* (**Figure 1L**; **Supplementary Figure 11**), nor did we notice a decrease in fruit set or the number of leaves due to reduction of leaf formation and growth. This could be attributed to the functional redundancy between tomato *ER* and its homologous gene *ERL* in regulating stomatal development. Compared to the study conducted by Kwon et al. (2020) we did observe additional phenotypes, such as an increase in the number of flowers and fruits in the mutant varieties. Considering the different phenotypes observed in *er* mutants caused by different mutation sites reported in *Arabidopsis* (Kosentka et al., 2017), we speculate that this difference may be attributed to different genetic backgrounds and mutation sites. These mutants are very important for exploring the function and mechanism of tomato.

### 4.2 ER is involved in the biosynthesis and signal transduction pathways of gibberellin

Numerous studies (Qu et al., 2017; Cai et al., 2020; Yang et al., 2020; Xu et al., 2023) have revealed a strong correlation between the inhibition of cell elongation observed in *ER* mutants and disturbances in auxin synthesis and transport processes. The *er erl1 erl2* triple mutant exhibited lower levels of auxin, accompanied by a significant decrease in the expression levels of key auxin biosynthesis genes. However, we did not observe a decrease in indol-3-acetic acid (IAA) levels in *sip* mutants (**Supplementary Figure 10B**). Is this possibly caused by abnormalities in auxin transport processes? We examined the expression levels of the *PIN* gene family, which is involved in auxin transport, in the stems of the mutant plants. The expression levels of these genes showed no difference compared to the WT plants (**Supplementary Figure 10C**). Instead, we observed significant changes in the gibberellin content, including a slight increase in GA_1_ and trace amounts of GA_3_ and GA_4_ (**Figure 5A**). External application of IAA did not restore the height of *sip* mutants to that of WT plants (**Supplementary Figure 10A**), whereas exogenous GA application did (**Figures 5E, H**). According to reports on gibberellin biosynthesis pathway, the intermediate product GA_12_-aldehyde can be converted into GA_12_ and GA_53_ through non-hydroxylation and hydroxylation pathways (Helliwell et al., 2001). GA_53_ is further catalyzed by a series of GA2 oxidases (GA2oxs) to form GA_20_. GA_20_ can then be degraded into GA_29_ by the action of GA2oxs or converted into GA_5_ by the catalysis of GA3oxs. GA_5_ subsequently undergoes further modifications to form the active gibberellin, GA_3_ (Yamaguchi, 2008). We found an increase in GA_29_ and a decrease in GA_5_ in *sip* (**Figure 5D**), which is consistent with the up-regulation of *GA2oxs* expression (**Figure 5C**). The increased GA_29_ suggests that more GA_20_ is being formed as a substrate, while the decreased GA_5_ and GA_3_ indicate a reduction in their synthesis from GA_20_ catalyzed by GA3oxs. These findings suggest that ER may regulate the gibberellin pathway by modulating the expression of GA_2_ oxidases.

Because of the presence of an extracellular LRR domain in ER, it is expected that there are ligands that can bind to the LRR domain. The protein kinase domains of ER in different species are relatively conserved, whereas the transmembrane and extracellular domains show less conservation (Kosentka et al., 2017). This suggests that there may be different ligands and co-receptors in different species but that they all likely utilize MAPK cascades as downstream pathways. ERf receptor activity is regulated by a group of cysteine-rich peptides belonging to the EPF/EPFL family (Hara et al., 2007; Shpak, 2013). More than 11 ligands have been identified in *Arabidopsis*, including members of the EFL family and TOOMANY MOUTHS (TMM) (Berger and Altmann, 2000). However, there are few genes with high homology to these secreted peptides in tomatoes. We hypothesized that different structural domains of ER may contain distinct ligand binding sites to perform their respective functions. The mutation N134I may result in the preventing the binding of ligands or reducing their activity, thus inhibiting the activation of the MAPK-mediated pathway that regulates plant height. Then, we performed MD simulations to study the folding of SlSIP and found that the mutated site could form hydrogen bonds with other internal amino acids. The alteration of N134I leads to changes in the hydrogen bonds formed within this particular site (**Figures 3C, D**), which provides support for this hypothesis.

In *Arabidopsis*, it has been reported that the ER-MAPK module regulates plant height by controlling the activity of DELLA through KNAT1/BREVIPEDICELLUS(BP) (Milhinhos et al., 2019). However, there are no homologous genes of *BP* in tomato, we speculate that there may be similar transcription factors that directly or indirectly regulate the expression of *GA2oxs* downstream of the ER-MAPK module in tomato. Through transcriptomic analysis, we have identified several differentially expressed transcription factors, including members of the MYB, WAKY, and MADS families. Members of these families reportedly participate in regulating inflorescence architecture and plant growth in *Arabidopsis*. We predicted whether the transcription factors from the differential expression can bind to the promoters of gibberellin-related genes. We found that Solyc10g005080 (MYB family) and MADS3 have binding sites for *ent-kaurenoic acid oxidase 1-like*, *GA2ox5*, *gibberellin-regulated protein 6*, *gibberellin-regulated protein 11*, *CPS*. This suggests that they may act as downstream regulators of the ER-MAPK module. These speculations and the mechanism of ER regulation of gibberellin need to be further elucidated in future studies.

### 4.3 *ER* mutant varieties offer potential for breeding strategies

ER is a receptor-like protein kinase of leucine-rich repeat RLKs that regulates diverse biological processes during plant growth and development, including seed germination, stem elongation, stomatal development, and inflorescence architecture. ER is involved in various functions, and its regulatory mechanisms are highly complex. There is extensive research on ER in *Arabidopsis*, but the research currently on ER in tomato is very limited. In *Arabidopsis*, four phosphorylation sites Thr^807^, Thr^812^, Tyr^815^, and Tyr^820^ are critical for the functionality of the ER kinase domain. Thr^807^ and Thr^812^ have a positive effect on ER function, whereas phosphorylation of Tyr^815^ and Tyr^820^ at these sites has an inhibitory effect on ER function (Kosentka et al., 2017). This suggests that ER signal transduction varies across different regulatory pathways, which explains the significant differences in the phenotypes observed among the different ER mutants. Interestingly, we compared the *sip* mutant and the knockout mutant *er*, and found that they exhibit consistent phenotypes in terms of internode length, inflorescence structure, fruit morphology, and so on. However, the mutations in sip still retain some of the functions of *ER*.

In *Arabidopsis*, Wu et al. isolated three *er* mutants, *er563*, *er795*, and *er1214*, containing 124, 99, and 632 amino acids, respectively (Wu et al., 2022). The seeds of *er563* and *er795* mutants are significantly smaller than those of WT, while *er1214* mutant shows normal seed size and weight. Only *er1214* contains intact extracellular and transmembrane domains but lacks the kinase domain. Consistent with these findings, we observed the same phenomenon in *sip* mutants and *er#15*,*16* lines. SlSIP did not affect seed size (**Figure 4E**), we speculate that this is because the kinase domain in SlSIP is not disrupted. Additionally, we found that if all inflorescences, including those generated from lateral branches, are retained, *sip* has more inflorescences compared to *er* (**Supplementary Figures 1J, 5C**). It seems that *sip* has a stronger ability to differentiate floral buds from lateral branches compared to *er*. Considering the previously reported role of ER in stress responses, we subjected *sip* to drought and cold stress, and it showed stronger tolerance compared to WT (not shown in this article). These findings suggest that *sip* carries a novel mutation in tomato that could separate this compact structure from its other regulatory functions.

We observe that both *sip* and *er* can produce more fruits, which is undoubtedly beneficial for production. Whether it is compact plant type or producing more fruits, it can be applied in tomato breeding. Concentrated flowering and fruiting are significant for processing tomatoes in production, as mechanical harvesting requires concentrated flowering and fruiting to adapt. As ER serves as an upstream central signal for plant growth and development (Shpak, 2013), and considering its role in response to both biotic (Godiard et al., 2003) and abiotic stresses (Du et al., 2018; Juneidi et al., 2020), would knocking out ER potentially have any adverse effects in production? The mutants like *sip* have more potential for application in breeding, it provides a possibility to separate the plant height and the ability to produce more fruits from other ER-controlled traits in tomato breeding.

In this study, we identified a shortened internodes and pedicel mutant *sip*, and conducted mapping through BSA analysis. The mutation that separates plant height from other traits controlled by ER, this separation allows for a combination of favorable traits, and providing a valuable foundation for a more thorough examination of ER function in tomato. These findings contribute to optimized breeding strategies for cultivating tomato varieties with desirable dwarfing traits and can serve as a reference for further investigations and applications in crop improvement and breeding programs targeting plant architecture and yield potential.

## Data availability statement

The datasets presented in this study can be found in online repositories. The names of the repository/repositories and accession number(s) can be found in the article/**Supplementary Material**.

## Author contributions

XZ designed and carried out the experiments and wrote the manuscript. KZ, HZ, MB analyzed the results, YH, YC, TC provided scientific advice, and revised the manuscript. MQ and JM conceived the research area, provided scientific advice, and supervised the project. All authors read and approved the final manuscript.
