# Supplementary material for: Tomato short internodes and pedicels encode an LRR receptor-like serine/threonine-protein kinase ERECTA regulating stem elongation through modulating gibberellin metabolism
Source: Front Plant Sci. 2023 Nov 17;14:1283489. doi: 10.3389/fpls.2023.1283489 (PMC10704031; doi:10.3389/fpls.2023.1283489)
Supplement: Supplementary file 1 [file DataSheet_1.docx]

**Tomato *short internodes and pedicels*(*SlSIP*) encode an LRR receptor-like serine/threonine-protein kinase ERECTA regulating stem elongation through modulating gibberellin metabolism**

**Xueya Zhao^1,2,3,4,5^, Kunpeng Zhang^1,2,3,4,5^, Huidong Zhang^1,2,3,4,5^, Mengxi Bi^1,2,3,4,5^, Yi He^1,2^, Yiqing Cui^1,2^, Changhua Tan^1,2,3,4,5^, Jian Ma^1,2,3,4,5*^ and Mingfang Qi^1,2,3,4,5*^**

^1^ College of Horticulture, Shenyang Agricultural University, Shenyang, China

^2^ National & Local Joint Engineering Research Center of Northern Horticultural Facilities Design &Application Technology (Liaoning), Shenyang, China

^3^ Key Laboratory of Protected Horticulture of Ministry of Education, Shenyang, China

^4^ Key Laboratory of Horticultural Equipment, Ministry of Agriculture and Rural Affairs, Shenyang, China

^5^ Collaborative Innovation Center of Protected Vegetable Provincial Co-construction Surrounds Bohai Gulf Region, Shenyang, China

*** Correspondence:**Mingfang Qi and Jian Ma
[qimingfang@126.com](mailto:qimingfang@126.com) and [mj76@163.com](mailto:mj76@163.com)


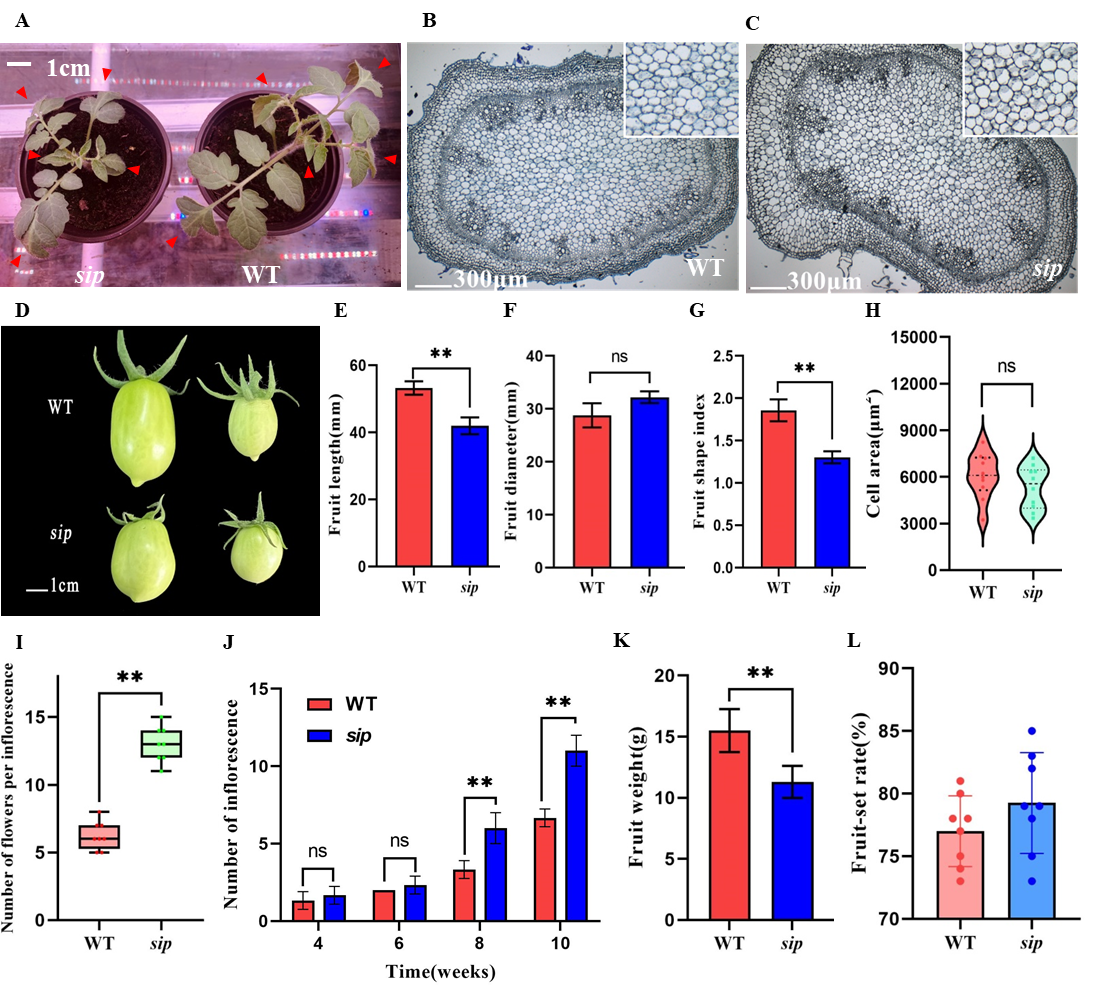


**Supplementary Figure 1** **Phenotypic identification of *sip*. (A)** Comparison of leaf blades between WT and *sip* seedlings. **(B, C)** Paraffin sections of the cross-sections of the third internode of stems in WT and *sip* seedlings during the fifth leaf stage. **(D)** Comparison of fruits morphology between WT and *sip* (first fruit of the first panicle in each, and first fruit of the second panicle in each). **(E)** Fruit length. **(F)** Fruit diameter. **(G)** Fruit shape index (ratio of fruit diameter to fruit length). **(H)** Comparison of the cell area at the central position in B and C. **(I)** The number of flowers per inflorescence in the primary spike at 40 days of seedling age. **(J)** Number of inflorescences of WT and *sip*. **(K)** Fruit weight (first fruit of the second panicle). **(L)** Fruit set rate in mature plants of WT and *sip*.


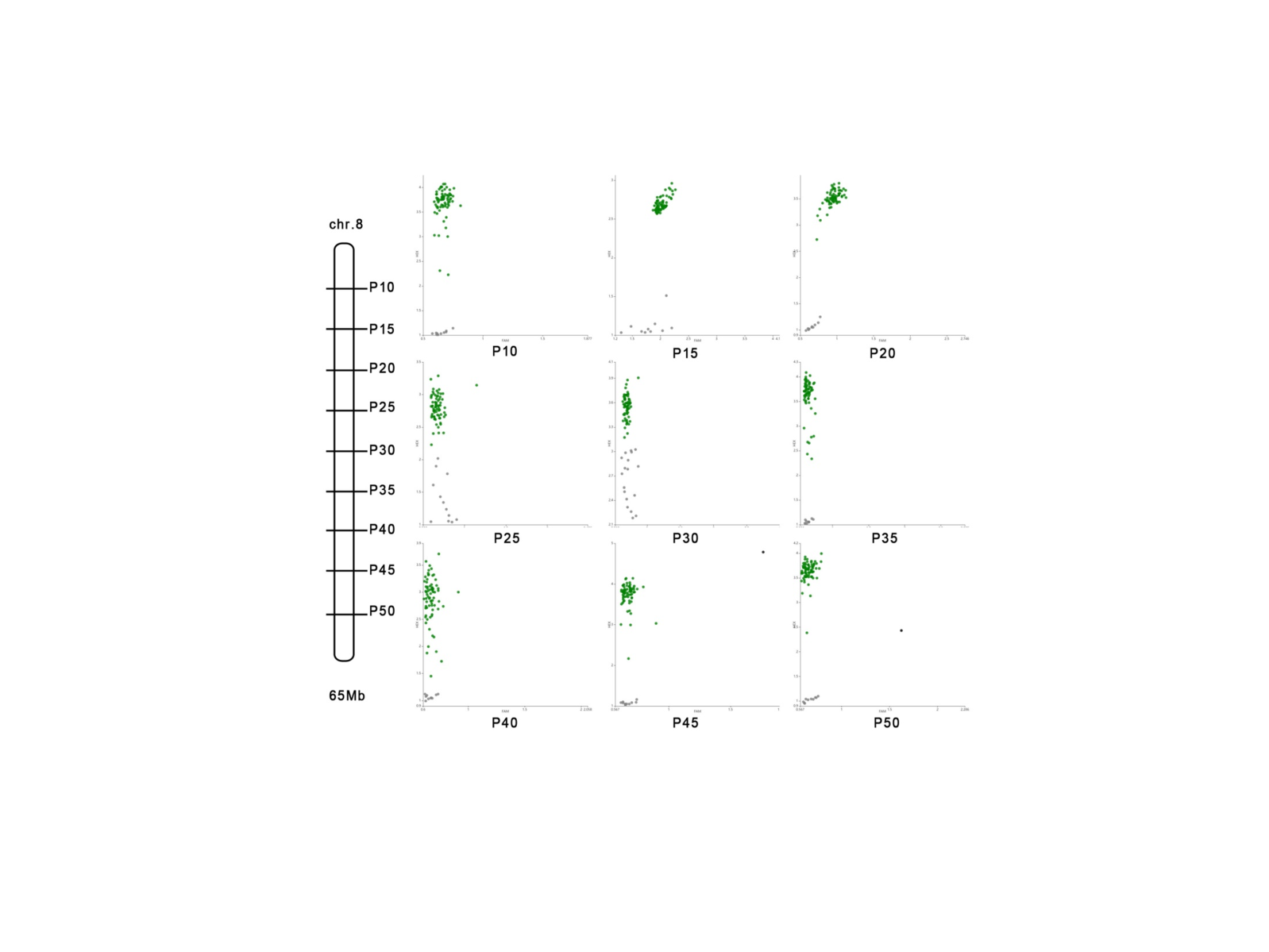


**Supplemental Figure 2** **PARMS genotyping in F2-*sip* (WT×*sip*) individuals.** The distribution of markers P10-P50 on chromosome 8 is depicted. The genotypes of marker P10-P50 in 74 *sip* individuals from the F2 population are represented. Genotypes with a HEX-type allele are colored blue as same with WT, while those with a FAM-type allele are colored green as same with *sip*. Heterozygous genotypes are shown in red.


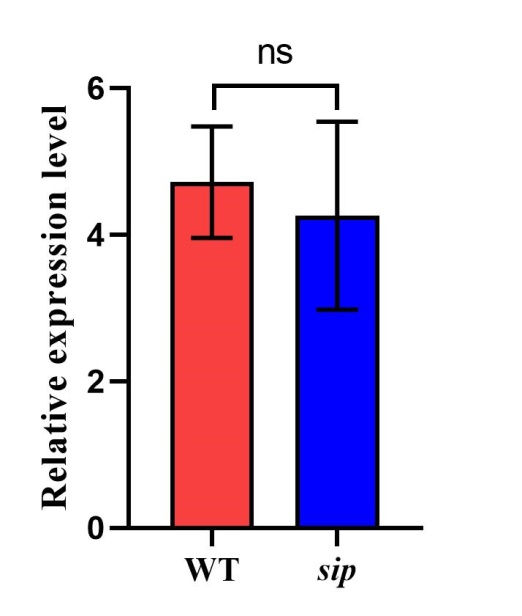


**Supplemental Figure 3 The relative expression level of *Solyc08g044280* in the stems of *sip* and WT at seedling stage.**


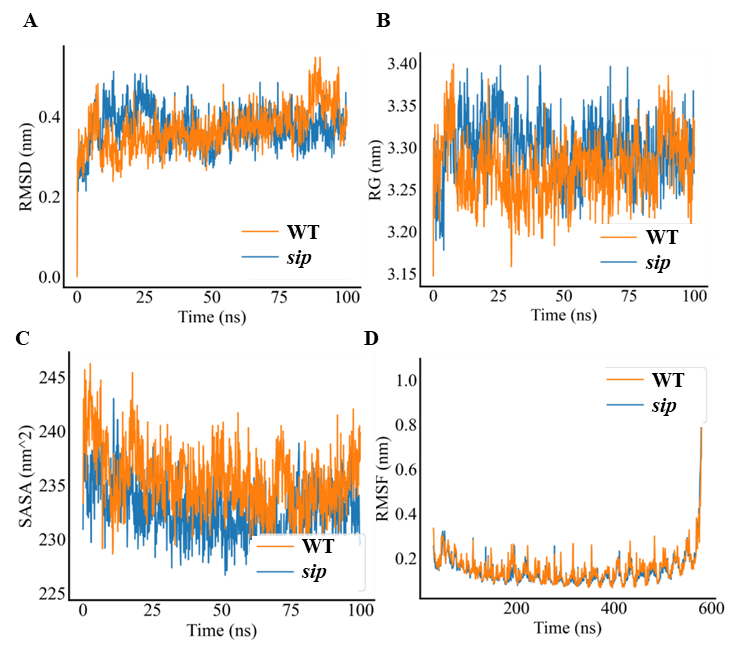


**Supplemental Figure 4** **MD simulations results for ER and SlSIP.** **(A)** Root Mean Square Deviation (RMSD) of the complex MD simulations. **(B)** Radius of Gyration (RoG). **(C)** Solvent-accessibility surface area (SASA). **(D)** Root Mean Square Fluctuation (RMSF).


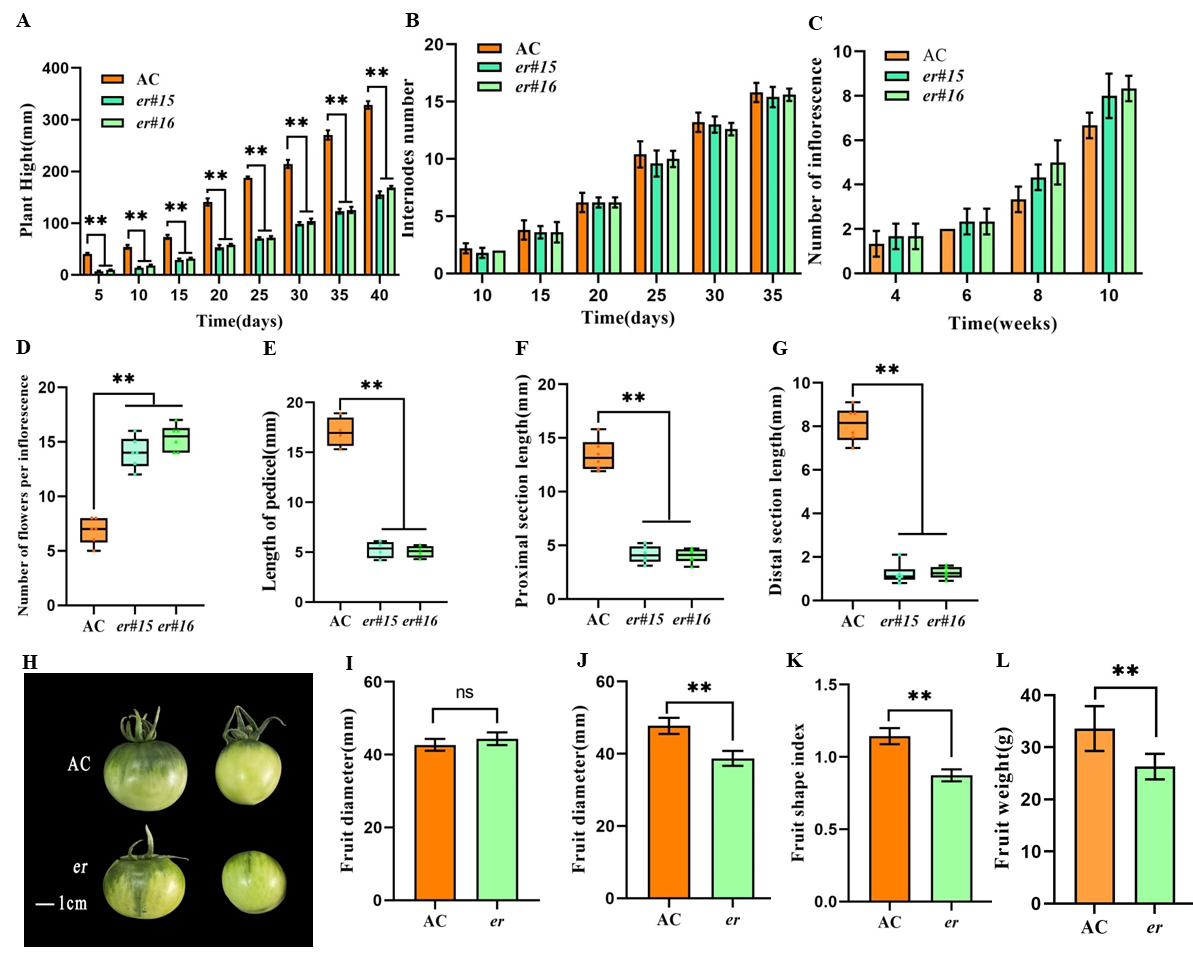


**Supplemental Figure 5** **Phenotypic identification of *sip*. (A, B)** Plant height and stem internode number of AC and *er* mutants at different stages (the beginning of sowing is regarded as 0 days, n=3). **(C)** Total number of inflorescences in AC and *er* mutant plants. **(D)** The number of flowers per inflorescence in the primary spike. **(E-G)** Length of the pedicels, lengths of the proximal section and distal section in AC and *er*. **(H)** Comparison of fruits morphology between AC and *er* (first fruit of the first panicle in each, and first fruit of the second panicle in each). **(I)** Fruit length. **(J)** Fruit diameter. **(K)** Fruit shape index (ratio of fruit diameter to fruit length). **(L)** Fruit weight.


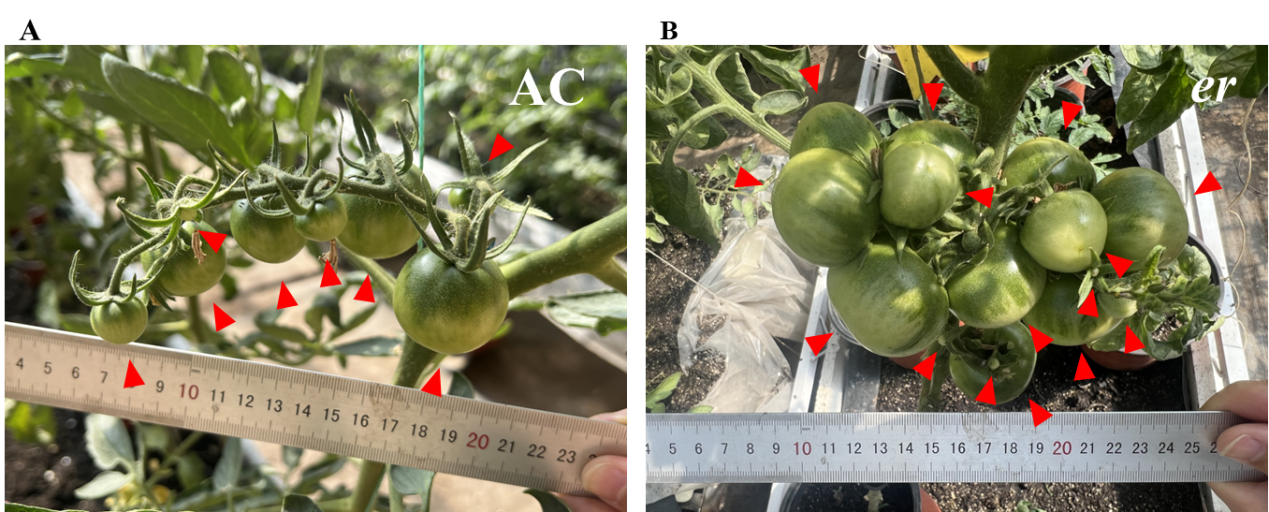


**Supplemental Figure 6** **Comparison of the fruiting status of individual inflorescences between AC and *er* mutant plants (first panicle).**


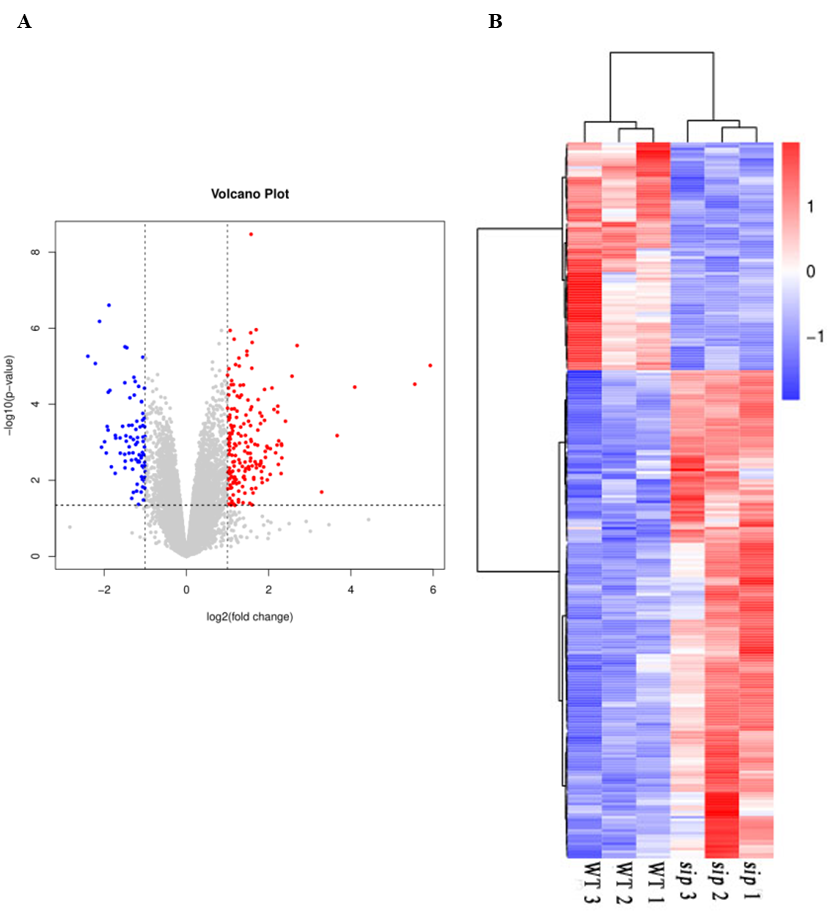


**Supplemental Figure 7 Volcanic map and Cluster map of differential genes. (A)** Volcano plot of gene expression in WT and *sip*. **(B)** Cluster map of differential genes.


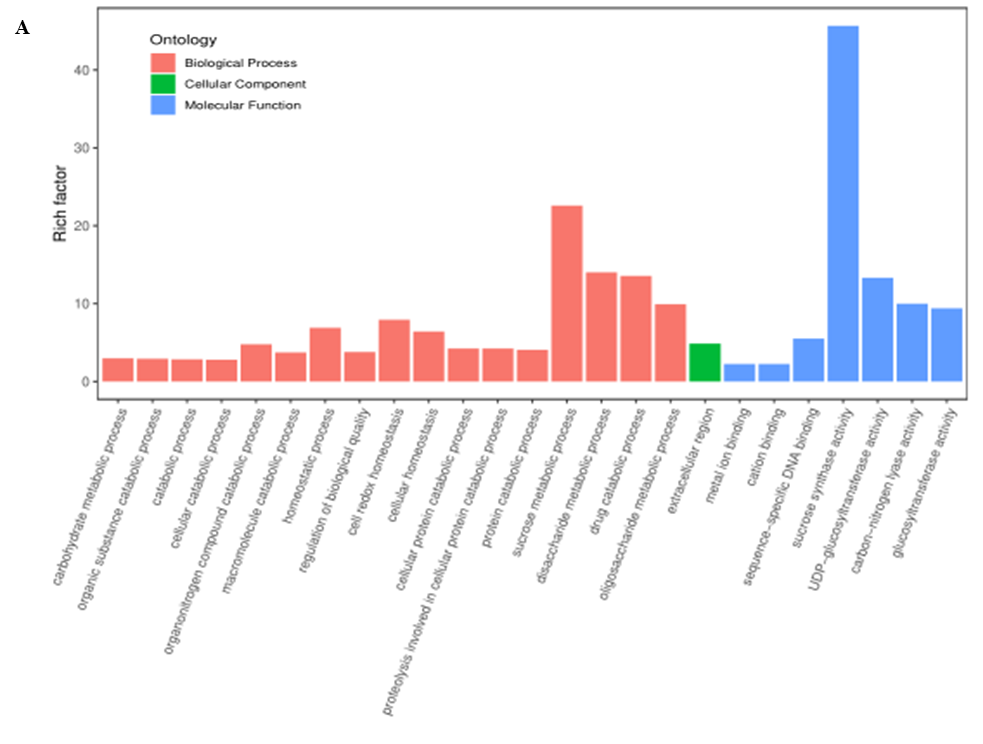


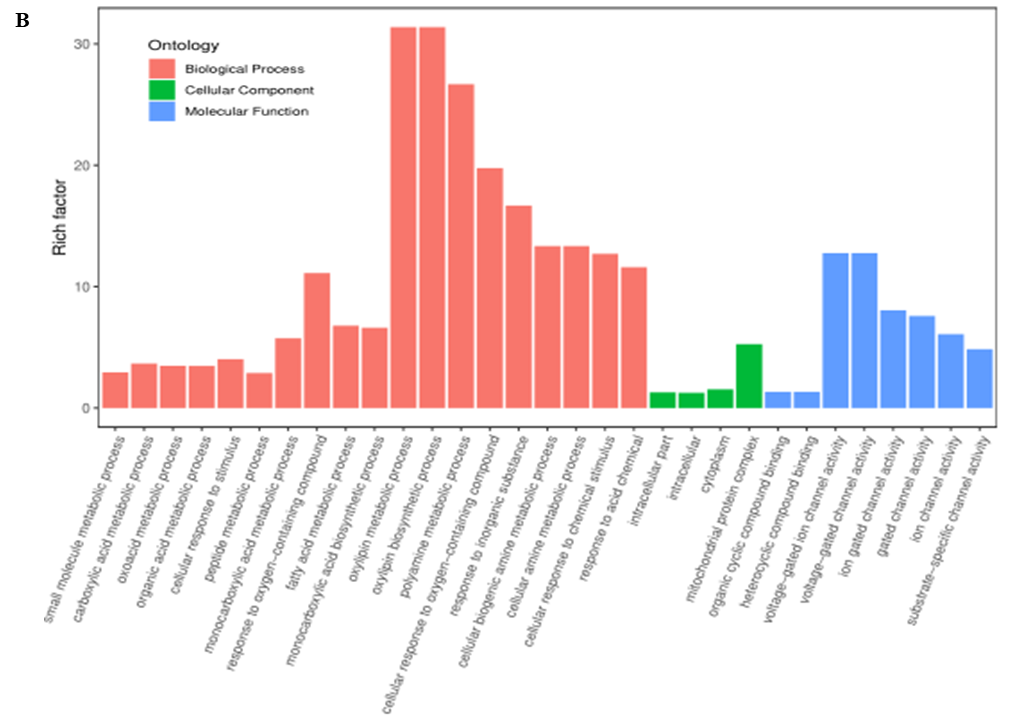


**Supplemental Figure 8 GO enrichment analysis. (A)** GO enrichment of up-regulated genes. **(B)** GO enrichment of down-regulated genes.


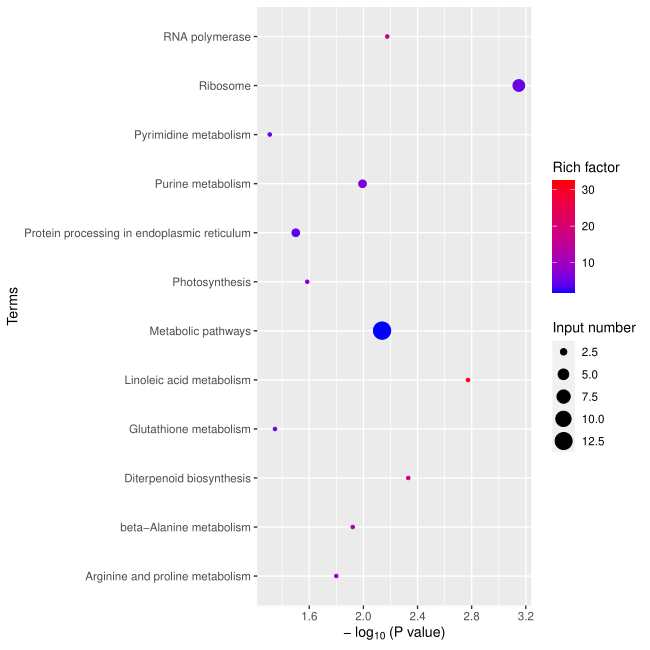


**Supplemental Figure 9 KEGG pathway map of down-regulated genes**


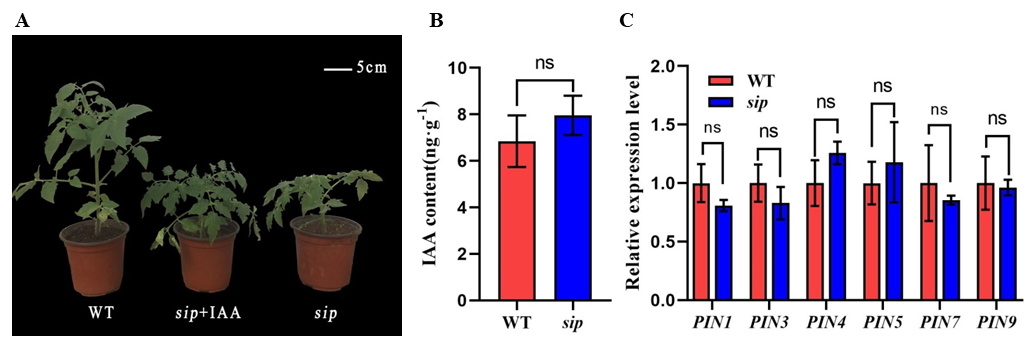


**Supplemental Figure 10** **IAA treatment and content levels of IAA in the young stems of *sip* mutants and WT. (A)** IAA treatment on *sip*. **(B)** IAA content levels in the young stems of *sip* mutants and WT (Take the third internode of the stem as the sample during the five-leaf stage). **(C)** The expression levels of the PIN family genes in the stems of WT and *sip*.

**
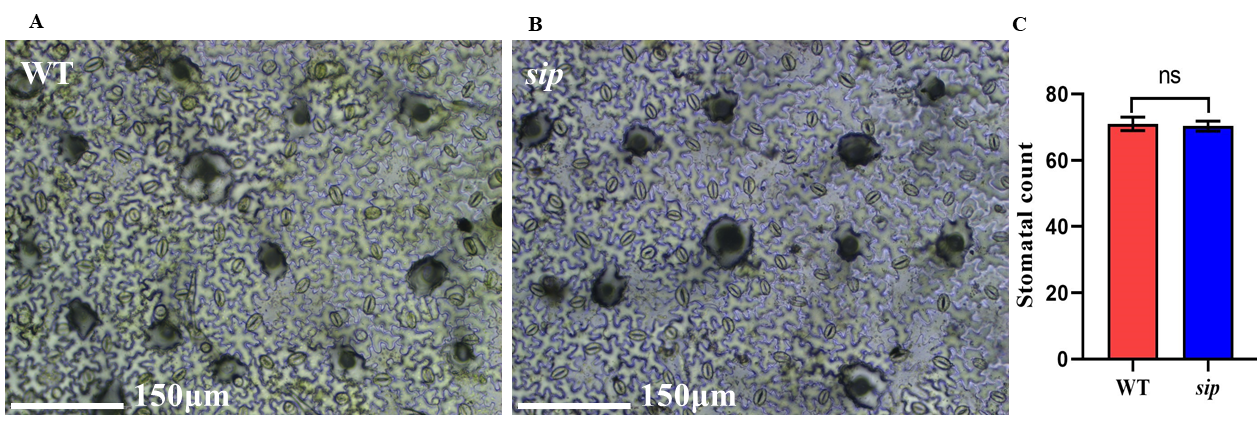
**

**Supplemental Figure 11** **Comparison of stomata number in WT and *sip* leaves. (A, B)** Microscopic observation of leaf stomata in WT and *sip*. **(B)** Statistical analysis of stomatal density in the field of view area.

**Table S1 Sequences of all primers for makers in this work**

| Markers name | Marker location | Primer sequence(5’-3’) |
| --- | --- | --- |
| P1 | 1173580 | Rc: GAAGGTGACCAAGTTCATGCTTAGCTAAAAGTAGATTGTGGATTGTAAC  Rt: GAAGGTCGGAGTCAACGGATTATAGCTAAAAGTAGATTGTGGATTGTAAT  F: AATTCGGCATATAAGTAAACATATACTAAC |
| P3 | 3204201 | Fg: GAAGGTGACCAAGTTCATGCTCCCAGTGCATAAAGCATCTCATG  Ft: GAAGGTCGGAGTCAACGGATTCCCAGTGCATAAAGCATCTCATT  R: CTTGCATTATGATAGGCTGTCTACATT |
| P10 | 10077285 | Rc: GAAGGTGACCAAGTTCATGCTTCTGAAATGAAATGTTAGGGACAAC  Rt: GAAGGTCGGAGTCAACGGATTTCTGAAATGAAATGTTAGGGACAAT  F: GCGGTTTGGATTAGCGATCTT |
| P15 | 15207312 | Fc: GAAGGTGACCAAGTTCATGCTCTGCTGATTGGGAGAGCTCC  Fa: GAAGGTCGGAGTCAACGGATTCTGCTGATTGGGAGAGCTCA  R: GTTATGTACCAAAACGACTGGGA |
| P20 | 20013839 | Rt: GAAGGTGACCAAGTTCATGCTTGCTGAAGGAAGATGGGGAAGT  Rc: GAAGGTCGGAGTCAACGGATTGCTGAAGGAAGATGGGGAAGC  F: GGGTGCTGGGATCACTTCTCA |
| P25 | 25259139 | Rc: GAAGGTGACCAAGTTCATGCTCCTCTCACCCTAAGTTAGTGATACCC  Rt: GAAGGTCGGAGTCAACGGATTACCTCTCACCCTAAGTTAGTGATACCT  F: TATCGCCAACTGAATAAAGTCACTATA |
| P30 | 30045905 | Fc: GAAGGTGACCAAGTTCATGCTGCATAATGTCATCAAATCACAAACC  Ft: GAAGGTCGGAGTCAACGGATTAGCATAATGTCATCAAATCACAAACT  F: TGATTACTTAAGGGGTTTGACTTAGTT |
| P35 | 35119359 | Fg: GAAGGTGACCAAGTTCATGCTGTTCCACCAGGGCAACAAG  Fa: GAAGGTCGGAGTCAACGGATTGTTCCACCAGGGCAACAAA  R: TGGGGATCCATAGGAAGTACCTA |
| P40 | 40302567 | Fg: GAAGGTGACCAAGTTCATGCTTTTTCTGCTAAAATTAACGATGAAG  Fa: GAAGGTCGGAGTCAACGGATTTTTTCTGCTAAAATTAACGATGAAA  R: CATAATCGAAATCTAGACCCCAA |
| P45 | 45223347 | Rt: GAAGGTGACCAAGTTCATGCTATATTGACACCTTGGTGAATTTATTT  Ra: GAAGGTCGGAGTCAACGGATTAATATTGACACCTTGGTGAATTTATTA  F: GTGATTGATTCTGTGATAGAGTTAGAGA |
| P50 | 50160562 | Rc: GAAGGTGACCAAGTTCATGCTCCTCACAAACTGAGTAAGAATTTGTC  Ra: GAAGGTCGGAGTCAACGGATTACCTCACAAACTGAGTAAGAATTTGTA  F: AATTAATACAGTTATGCATGCTAGAACA |
| P55 | 55815482 | Fg: GAAGGTGACCAAGTTCATGCTATAACAGTTAAGGTCTGGGGCAG  Fa: GAAGGTCGGAGTCAACGGATTATAACAGTTAAGGTCTGGGGCAA  R: AGCTCGGGATTGACTTTAATGTG |
| P61 | 61199142 | Ft: GAAGGTGACCAAGTTCATGCTAATTCAAAATCACCAGATTTTCGT  Fc: GAAGGTCGGAGTCAACGGATTATTCAAAATCACCAGATTTTCGC  R: TCGAAGTATCAGGTGCATTATGG |
| P63 | 63193607 | Fg: GAAGGTGACCAAGTTCATGCTTAATCGTCAAGCTTGTTGATATCTG  Fa: GAAGGTCGGAGTCAACGGATTTTAATCGTCAAGCTTGTTGATATCTA  R: GCCCTTTTAGAGCCTCAGAATA |
| M20 | 21869137 | F1: GAAGGTCGGAGTCAACGGATTAAATGACGGGCTTAGCGTTA  F2: GAAGGTGACCAAGTTCATGCTAATGACGGGCTTAGCGTTG  R: GCTCCACCTTGTTGACACCC |
| M40 | 41250405 | F1: GAAGGTCGGAGTCAACGGATTGCACAGTCTCCAACAGTGGTAA  F2: GAAGGTGACCAAGTTCATGCTGCACAGTCTCCAACAGTGGTAG  R: CCCATTGGCTGAGGTTTCT |
| K1 | 47106679 | F1: GAAGGTCGGAGTCAACGGATTCCTTAGGGACCTTTCCTTCAT  F2: GAAGGTGACCAAGTTCATGCTCCTTAGGGACCTTTCCTTCAA  R: TGAGTTTAGATATGGAGAAGGGAAT |
| K2 | 49603202 | F1: GAAGGTCGGAGTCAACGGATTCACATTTCCTTTCCGTTTCC  F2: GAAGGTGACCAAGTTCATGCTCCACATTTCCTTTCCGTTTCT  R: TTTCACTTTGGGCTATTAGGTTG |

**Table S2 Sequences of all primers for qRT-PCR in this work**

| Gene ID | Gene name | Primer sequence(5’-3’) |
| --- | --- | --- |
| *Solyc03g078400* | *SlACTIN* | F: TGTCCCTATTTACGAGGGTTATGC  R: AGTTAAATCACGACCAGCAAGAT |
| *Solyc08g015900* | - | F: CACCCCCTTCGGATACACCT  R: AAGCCTCCTCGTCTCTTCGT |
| *Solyc08g044280* | - | F: GTGAGTCACTACATCGCCAGAGC  R: TCCAACAACTTCGAACAGCAT |
| *Solyc08g047990* | - | F: TGGGGGTGAAAATGAAGTTGAT  R: TCCGAGTTGTACTGATGATGGTT |
| *Solyc05g053340* | *GA2ox1* | F: ATGTAAAATCAGTGAGGGAGTTGG  R: CGTAGTAGAGAATCAGAATGAAGGTC |
| *Solyc07g056670* | *GA2ox2* | F: CTAAACCCGACTCCAAGAACC  R: TTATTGCCATAGCCAAAAGGAT |
| *Solyc01g079200* | *GA2ox3* | F: GGCTGAAGGTTTATGGATAGAAGA  R: CGAAGGATTGTTAGTATTTGTGGA |
| *Solyc07g061720* | *GA2ox4* | F: CCTGCCATTACTCACTTCCCT  R: CCCTGCCTTTAGTTTCTCAGAG |
| *Solyc07g061730* | *GA2ox5* | F: CAATAAACAAATCGGACAAAGTGG  R: CATGGAGGATAATGATTCAGCCT |
| *Solyc02g080120* | *GA2ox6* | F: TTAGCCACCTCCACTTCTCAAT  R: TCATCCCTGCTTATCTTTTGGT |
| *Solyc11g011260* | *GA2ox7* | F: GGTTTCAAATGCGTTCAAACAAG  R: GTATGCCACCCCAACATAAGAC |
| *LOC101266437* | *ER* | F: TCATTTGATTCTAACTAAGGCAGC  R: TTGTTGGTCTCTCAGCAGGC |
| *Solyc03g118740* | *PIN1* | F: AGTTGGCAAGGATCTTCAT  R: GCAAAGCTGAAATCATCTCTC |
| *Solyc07g006900* | *PIN2* | F: CAGTGCCAGTACCAGTAC  R: CGAACTTGCATTCTCTCTTG |
| *Solyc04g007690* | *PIN3* | F: TTCAAAATCAATTTAGCGTGTCA  R: CTCAAAATCCCTCTTGTTTCG |
| *Solyc05g008060* | *PIN4* | F: TGGTGTTGCAGTGTATCATTTGG  R: TCGCACCGCGATATTCG |
| *Solyc01g068410* | *PIN5* | F: ACATTGAGCTGGCATTTTGG  R: TCCACTACCAGCCTTTGACA |
| *Solyc06g059730* | *PIN6* | F: AGATGGCAGCAATAGGGATG  R: GCGAAGACAAATGGAACGAT |
| *Solyc10g080880* | *PIN7* | F: ATCAGCGGTCCAGCAGTCAT  R: AAAGCCCCAGCAAAATGTAGTA |
| *Solyc02g087660* | *PIN8* | F: ACAACCTCCTCCTACTCCTATTCG  R:GCTTCCTCCTCACCGTCCTC |
| *Solyc10g078370* | *PIN9* | F:GTGGCGAAAACTAATCAGGAAC  R: CCATGAACAAACCAAGACTAAACA |
| *Solyc04g056620* | *PIN10* | F: ATTATGGCTTGTGAGGGTGCT  R: TTCCTGCCTTTGACATTATTGAT |

**Table S3 List of candidate genes base on QTL-seq**

| Candidate Genes | Pos | Ref | Alt | Function Type | Position |
| --- | --- | --- | --- | --- | --- |
| *Solyc08g061560* | 47106679 | T | A | nonsynonymous SNV | exon5:401 |
| *Solyc08g062600* | 49603202 | G | A | nonsynonymous SNV | exon1:343 |
| *Solyc08g048250* | 14004482 | G | A | - | intron5:890 |
| *Solyc08g022130* | 31865227 | G | A | - | intron3:342 |
| *Solyc08g029380* | 35116047 | T | A | - | intron3:84 |
| *Solyc08g028690* | 41250405 | G | A | - | intron1:8357 |
| *Solyc08g061500* | 46933486 | G | A | - | intron23:21 |
| *Solyc08g065870* | 52310931 | G | A | - | intron3:1444 |
| *Solyc08g044337* | 21206973 | G | A | - | upstream dist:249 |
| *Solyc08g015900* | 6189225 | C | T | - | upstream dist:976 |
| *Solyc08g066330* | 52965279 | C | A | - | upstream dist:470 |
| *Solyc08g047990* | 14912391 | C | A | - | downstream dist:781 |
| *Solyc08g044280* | 21869137 | G | A | - | downstream dist:381 |
| *Solyc08g036550* | 10658027 | G | A | - | downstream dist:122 |

Upstream: the region 2 kb upstream of the transcription start site. Downstream: the 2 kb region downstream of the transcription termination site.

**Table S4 KASP was labeled in F2 populations of WT and *sip* hybrids**

| Candidate Genes | M20 | M40 | K1 | K2 |
| --- | --- | --- | --- | --- |
| SNP position | 21869137 | 41250405 | 47106679 | 49603202 |
| WT | A | A | A | A |
|  | A | A | A | A |
|  | A | A | A | A |
| *sip* | B | B | B | B |
|  | B | B | B | B |
|  | B | B | B | B |
| F2 *sip* individuals | B | B | B | B |
|  | B | B | B | B |
|  | B | B | B | H |
|  | B | B | B | B |
|  | B | B | B | B |
|  | B | B | B | B |
|  | B | B | B | B |
|  | B | B | B | B |
|  | B | B | B | B |
|  | B | B | B | B |
|  | B | B | B | B |
|  | B | B | B | B |
|  | B | B | B | B |
|  | B | B | B | B |
|  | B | B | B | B |
|  | B | B | B | B |
|  | B | B | B | B |
|  | B | B | B | B |
|  | B | B | B | B |
|  | B | B | B | B |
|  | B | B | B | B |
|  | B | B | B | B |
|  | B | B | B | B |
|  | B | B | B | B |
|  | B | B | B | B |
|  | B | B | B | B |
|  | B | B | B | B |
|  | B | B | B | B |
|  | B | B | B | B |
|  | B | B | B | B |
|  | B | B | B | B |
|  | B | B | B | B |
|  | B | B | B | B |
|  | B | B | B | B |
|  | B | B | B | B |
|  | B | B | B | B |
|  | B | B | B | B |
|  | B | B | B | B |
|  | B | B | B | B |
|  | B | B | B | B |
|  | B | B | B | B |
|  | B | B | B | B |
|  | B | B | B | B |
|  | B | B | B | B |
|  | B | B | B | B |
|  | B | B | B | B |
|  | B | B | B | B |
|  | B | B | B | B |
|  | B | B | B | B |
|  | B | B | B | B |
|  | B | B | B | B |
|  | B | B | B | B |
|  | B | B | B | B |
|  | B | B | B | B |
|  | B | B | B | B |
|  | B | B | B | B |
|  | B | B | B | B |
|  | B | B | B | B |
|  | B | B | B | B |
|  | B | B | B | B |
|  | B | B | B | B |
|  | B | B | B | B |
|  | B | B | B | B |
|  | B | B | B | B |
|  | B | B | B | B |
|  | B | B | B | B |
|  | B | B | B | B |
|  | B | B | B | B |
|  | B | B | B | B |
|  | B | B | B | B |
|  | B | B | B | B |
|  | B | B | B | B |
|  | B | B | B | B |
|  | B | B | B | B |
|  | B | B | B | B |
|  | B | B | B | B |
|  | B | B | B | B |
|  | B | B | B | B |
|  | B | B | B | B |
|  | B | B | B | B |
|  | B | B | B | B |
|  | B | B | B | B |
|  | B | B | B | B |
|  | B | B | B | B |
|  | B | B | B | B |
|  | B | B | B | B |
|  | B | B | B | B |
|  | B | B | B | B |
|  | B | B | B | B |
|  | B | B | B | B |
|  | B | B | B | B |
|  | B | B | B | B |

A genotype of tall plants, B genotype of plants with short internodes and pedicels, and H genotype of heterozygous plants
